# Testing assembly strategies of *Francisella tularensis* genomes to infer an evolutionary conservation analysis of genomic structures

**DOI:** 10.1186/s12864-021-08115-x

**Published:** 2021-11-14

**Authors:** Kerstin Neubert, Eric Zuchantke, Robert Maximilian Leidenfrost, Roebbe Wuenschiers, Josephine Grützke, Burkhard Malorny, Holger Brendebach, Sascha Al Dahouk, Timo Homeier, Helmut Hotzel, Knut Reinert, Herbert Tomaso, Anne Busch

**Affiliations:** 1 Department of Mathematics and Computer Science, Algorithmic Bioinformatics, Freie Universität Berlin, Institute of Computer Science, Takustr. 9, 14195 Berlin, Germany; 2 German Federal Institute for Risk Assessment, Diedersdorfer Weg 1, 12277 Berlin, Germany; 3 Friedrich-Loeffler-Institut, Institute of Bacterial Infections and Zoonoses, Naumburger Str. 96a, 07749 Jena, Germany; 4 Department of Biotechnology and Chemistry, Mittweida University of Applied Sciences, Technikumplatz 17a, 09648 Mittweida, Germany; 5 Friedrich-Loeffler-Institut, Institute of Epidemiology, Südufer, 10 17493 Greifswald, Insel Riems Germany; 6 Department of Anaesthesiology and Intensive Care Medicine, University Hospital Jena, Jena, Germany

**Keywords:** *Francisella* pathogenicity island, Insertion sequences, High-throughput sequencing, Short-read assembly, Hybrid assembly, Ion Torrent’s ion S5, Illumina MiSeq, Illumina HiSeq; Pacific biosciences RS, Oxford Nanopore technologies MinION

## Abstract

**Background:**

We benchmarked sequencing technology and assembly strategies for short-read, long-read, and hybrid assemblers in respect to correctness, contiguity, and completeness of assemblies in genomes of *Francisella tularensis*. Benchmarking allowed in-depth analyses of genomic structures of the Francisella pathogenicity islands and insertion sequences. Five major high-throughput sequencing technologies were applied, including next-generation “short-read” and third-generation “long-read” sequencing methods.

**Results:**

We focused on short-read assemblers, hybrid assemblers, and analysis of the genomic structure with particular emphasis on insertion sequences and the Francisella pathogenicity island. The A5-miseq pipeline performed best for MiSeq data, Mira for Ion Torrent data, and ABySS for HiSeq data from eight short-read assembly methods. Two approaches were applied to benchmark long-read and hybrid assembly strategies: long-read-first assembly followed by correction with short reads (Canu/Pilon, Flye/Pilon) and short-read-first assembly along with scaffolding based on long reads (Unicyler, SPAdes). Hybrid assembly can resolve large repetitive regions best with a “long-read first” approach.

**Conclusions:**

Genomic structures of the *Francisella* pathogenicity islands frequently showed misassembly. Insertion sequences (IS) could be used to perform an evolutionary conservation analysis. A phylogenetic structure of insertion sequences and the evolution within the clades elucidated the clade structure of the highly conservative *F. tularensis*.

**Supplementary Information:**

The online version contains supplementary material available at 10.1186/s12864-021-08115-x.

## Background

*Francisella (F.) tularensis* is a highly infectious, Gram-negative, fastidious bacterial pathogen [1]. *F. tularensis* causes tularemia. It is considered a potential biological agent. *F. tularensis* subspecies *holarctica* is endemic in Europe [2]. For phylogenetic studies and outbreak analyses, high-quality reference genomes are needed [3, 4].

Studies of the genomic structure of *Francisella*, such as pathogenicity islands and insertion sequences, allowed new insights into the development of the species*.* Pathogenicity islands are defined as a class of genomic islands acquired by microorganisms through horizontal gene transfer and contribute to evolution. Insertion sequences (IS elements) are transposable elements, which code only for transposition activity and can occur in different copy numbers and positions within the genome [5, 6]. IS elements constitute genomic rearrangement events during evolution that are correlated to pathogenicity [7–9].

Genome sequencing is necessary to elucidate the genome structure and the phylogenetic and evolutionary context of bacteria. The analysis of bacteria with a very conservative genome structure, such as *F. tularensis*, requires reliable sequence data.

Short-read sequencing and long-read sequencing technologies can be used to exploit genomes. However, the reconstruction of genome assembly is complex, especially when it comes to duplications of genetic element as in *F. tularensis*. With *F. tularensis* subsp. *tularensis* strain SCHU S4 a Sanger sequenced reference genome is available, and assembly results could be evaluated and benchmarked by direct mapping [4].

Thus, we could evaluate de novo assembly methods for short-read, long-read and hybrid approaches. The most frequently used methods are based either on Overlap-Layout-Consensus graph, de Bruijn graph, or greedy approaches.

Assembly of reads into contigs with one of these approaches is followed by scaffolding contigs using mate-pair or paired-end reads.

The quality of the resulting assembly can be evaluated by comparing it to previously published finished genomes. For an evaluation, genome size, GC content, and repetitive regions were evaluated based on an independent reference genome created with a different method: Sanger sequencing as the gold standard. The accuracy of the assembly depends on sequencing technology, genomic structure, and used algorithms. Short-read technologies are accurate at low costs with low sequencing error rates. However, large duplications such as the 27 kb *Francisella* pathogenicity island (FPI) cannot be resolved solely by short-read assembly.

Consequently, most available bacterial genomes are incomplete or fragmented. Long reads can exceed the length of repeats and resolve repeats but have relatively high sequencing error rates. However, some library preparation techniques for Oxford Nanopore Technologies MinION sequencer (ONT) can perform poorly for genomes with low GC contents [10–15]. With a GC content of 32%, *Francisella* represents AT-rich pathogens that are far more frequent than GC-rich organisms [16], making them less prone to segregating mutations. As mutations occur only rarely, the analysis of their genomic nature can be challenging [17].

Hybrid assembly strategies combine the accuracy of short-read sequencing with the capacity to extend over long reads. The optimal combination of long and short-reads is unpredictable and needs to be assessed. We evaluated different assembly strategies and determined several quality parameters like error rates, contiguity, misassemblies, and the number of circularized contigs.

The *Francisella pathogenicity island (FPI)* is a gene cluster that occurs twice (duplicated) in the genomes of *F. tularensis* ssp. *holarctica* and *F. tularensis* ssp. *tularensis. F. tularensis* ssp. *novicida* has only one copy of this region [18, 19]*.* The function and expression of the FPI have already been studied extensively [7]. It is known that the FPI is challenging to resolve on a genomic level [7]. The FPI contains a cluster of 16–19 genes that encode the *Francisella* Type VI Secretion System, which is essential for pathogenicity [20].

We aimed to determine which sequencing technology suits best for analyzing *Francisella.* Although in this context, many studies are performed, few used Sanger sequenced genomes as a gold standard or used all major sequencing technologies [21]. The genome NC_006570.2 generated with Sanger sequencing was used as the reference genome [6]. Sanger Sequencing is time-consuming and thus expensive, but it produces relatively long DNA sequences of high quality.

We evaluated five independent sequencing technologies: Illumina MiSeq (MiSeq), Illumina HiSeq (HiSeq, and Ion Torrent’s Ion S5XL (Ion Torrent’s) to generate short-read sequences; Pacific Biosciences RS (PacBio) and Oxford Nanopore Technologies MinION (MinION) for long-read sequences. Sequencing technologies have been extensively described elsewhere [22]. We sequenced four genomes of diagnostically relevant *F. tularensis* subsp. *holarctica* from Germany together with *F. tularensis* subsp. *tularensis* SCHU S4 (FSC237). It is well known that each sequencing technology is prone to specific error types with different probabilities [23–27].

Short-read assemblies are less error-prone and cheaper. They are used frequently in diagnostic laboratories, where SNP detection and outbreak analysis are relevant. All three short-read sequencing technologies and eight free available software solutions [28–36] were assessed and evaluated. We benchmarked the optimal combination of sequencing technology and assembly software. The parameters for the evaluation of the assemblies were total length, GC content, assembly contiguity, error rate, genomic fraction, and gene annottion in accordance with established software solutions [37–42]. To achieve better insight into the assembler performance, we preprocessed reads either by downsampling or filtering  with a minimum length cutoff, respectively a quality cutoff. Assembly quality was assessed statistically and visually [43, 44].

The growing availability of 3rd generation sequencing technologies such as Single Molecule, Real-Time (SMRT) sequencing (PacBio), and nanopore MinION sequencing enable sequencing of long reads up to 65 kb or even up to several hundreds of kb in the latter case. Assembly of high-quality genomes can be achieved using exclusively long reads or short reads, preferably paired-end or mate-pair reads, to resolve complex genomic structures and compensate for the somewhat erroneous long reads.

Hybrid assemblers use two different sequencing technologies (long-reads and short-reads) to produce high-quality sequences [24, 37, 39,40,44, 45]. This approach improves scaffolding and makes the process computationally more efficient [21]. We assessed two-hybrid assembly strategies to establish optimal genome assemblies. The first approach is the “long-read first” approach as performed with Canu/Pilon and Flye/Pilon [46–48]. Long reads are assembled, and resulting assemblies are corrected with short reads. The second approach is the “short-read first” approach as performed with SPAdes and Unicycler [34, 49]. Therein short-reads are assembled into contigs, which are mapped to a long-read scaffold.

**Fig. 1:**
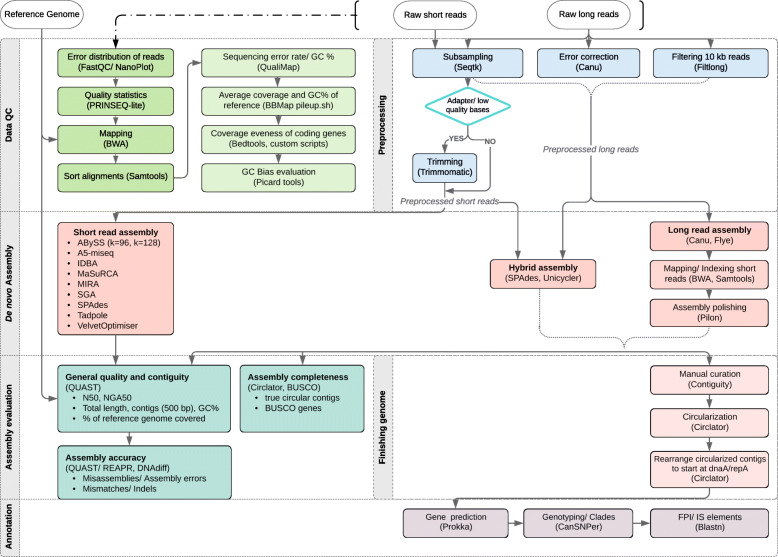
Processing workflow for sequencing data including data QC, preprocessing, de novo assembly, assembly evaluation and annotation (utilized tools in brackets).

We benchmarked with a processing workflow (Fig. 1) for an optimal combination of sequencing technology and assembly software that produces high-quality assemblies for elucidation of genome structure, including the FPI and the insertion sequences.

## Results

### Sequencing data quality

Illumina MiSeq sequencing yielded approximately 0.5 million paired-end reads with a median read length of 251 bp and 148–266 Mb data per isolate. Approximately 99.8% of reads could be mapped to the respective reference genome resulting in 99.2–99.7% of bases covered with 78-140x on average. The anticipated median insert size was 600 bp.

Illumina HiSeq sequencing yielded between 9 and 48 million paired-end reads with a length of 151 bp resulting in 2.7–17.7 Gb sequence data per isolate. Mapping to reference genomes resulted in 1432–7711x average coverage with all genomic positions covered. The anticipated median insert size was 300 bp.

Ion Torrent sequencing yielded 0.5–1.2 million single-end reads with a median length between 314 and 348 bp and a maximum of 533 bp, with 175–389 Mb data per sample. Reference genomes were covered 92-204x with around 100% bases covered.

PacBio SMRT sequencing data yielded 116,415–156,128 sub-reads per isolate with a median length of 4.4–7.5 kb and a maximum of 55.8 kb, generating 1–1.3 Gb data per isolate. Mapping to reference genomes resulted in 383-533x average coverage.

MinION sequencing yielded between 103,063 and 407,864 with a median length of 619–1317 bp and a maximum of 1.5 Mb. Between 86 and 90% of ONT-reads could be mapped to the reference genome resulting in a coverage of 64-282x with all bases covered at least once (Supplementary Table 1).

The long-read sequences were additionally assessed with a bivariate plot of the log-transformed read length against base-call quality with hexagonal bins and marginal histograms [41] (Supplementary Fig. 2).

The log-transformed read lengths of all data showed differences in the length profile with longer reads in the PacBio dataset. We observed a higher variability of the read length distribution across samples for MinION than PacBio (Supplementary Fig. 2).

#### Alignment validation and sequencing error

                                                                                                                                                                                                             The sequencing error rates could be assessed using an alignment validation with the reference genome of the isolate *F. tularensis* subsp. *tularensis* strain SCHU S4 (FSC237), NC_006570.2 (Table 1).

**Table 1:** Sequencing data and error rates for isolate FSC237 to reference NC_006570.2, with a GC content of 32.26%

Platform	GC reads (%)	Mapped bases (bp)	Mismatches	Insertions	Deletions	Mismatch error rate (%)	Insertions error rate (%)	Deletions error rate (%)	Total error rate (%)	Even score	Total error rate added (%)
MiSeq	36.29	265,564,803	710,772	653	8371	0.268	0.0002	0.0032	0.27	0.6715	0.2710
HiSeq	32.12	7,402,011,869	22,972,180	43,344	56,564	0.310	0.0006	0.0008	0.31	0.9862	0.3117
Ion Torrent	32.66	350,087,308	783,525	911,991	522,265	0.224	0.2605	0.1492	0.51	0.9400	0.6335
PacBio	32.45	1,118,125,280	77,755,474	50,861,188	35,660,450	6.954	4.5488	3.1893	14.99	0.9747	14.6922
MinION	32.26	533,513,044	68,465,798	13,287,287	18,136,808	12.833	2.4905	3.3995	16.88	0.9669	18.7230

ONT sequencing reads had the highest total error rate with 16.88%, followed by PacBio with 14.99%. MiSeq reads contained the lowest number of errors with only 0.27%, closely followed by HiSeq reads with 0.31% and Ion Torrent with 0.51%. We found the smallest percentage of insertions in MiSeq reads (0.0002%), but four times higher, percentage of deletions (0.0032%) compared to HiSeq (0.0008%). PacBio and Ion Torrent reads were more prone to insertions (4.55 and 0.26%), whereas ONT-reads were more susceptible to deletions (3.4%).

#### Coverage evenness and GC bias

We analyzed the sequencing bias of each technology by computing the distribution of coverage across protein-coding genes and 100-base windows with various GC percentages. The evenness score was very similar across technologies, with the best score for HiSeq (0.99) and PacBio (0.97), except for MiSeq with a relatively low score of 0.6715 (Table 1). The uneven coverage of MiSeq data was demonstrated in its biased coverage distribution with 85 genomic regions with low coverage < 15 and 302 regions with coverage between 15 and 45 (Supplementary Fig. 1) .

**Fig. 2:**
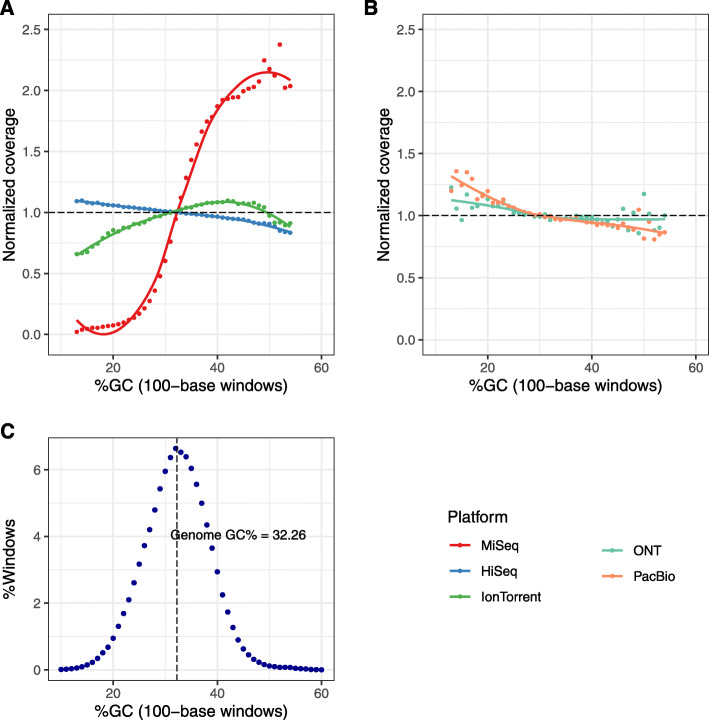
GC-bias plots for dataset FSC237 sequences for short-read (**A**) and long-read platforms (**B**). Normalized coverage is plotted for GC percentages with at least 1000 windows in the genome. Unbiased coverage is represented by a dashed line at normalized coverage of 1. GC distribution of FSC237 to reference NC_006570.2 (**C**)

GC percentage of MiSeq reads deviated with 36.29% substantially from the GC content of the reference genome with 32.3%, resulting in a strong bias towards reads with high GC content (Table 1, Fig. 2). While sequencing and base-call errors could be compensated with a slightly higher sequencing depth, GC bias had a pervasive impact on the result of de novo assembly and genotyping. Interestingly, normalized coverage of HiSeq, PacBio, and ONT was biased towards genomic regions with low GC content, while MiSeq and Ion Torrent sequencing preferred regions with high GC content (Fig. 2). We observed the lowest GC bias within short-read and long-read platforms for HiSeq and ONT, respectively.

### Evaluation of short-read assemblers

Long-read sequencing is error-prone and depends on high-quality DNA. In typical settings, short-read sequencing is mostly cheaper, more robust, and often used in diagnostics. We assessed the best short-read assembly strategy for *F. tularensis*. All short-read sequences of MiSeq, HiSeq, and Ion Torrent were evaluated with eight short-read assemblers: ABySS [28], A5-miseq [29], IDBA [30], MaSuRCA [31], MIRA [32], SGA [33], SPAdes [34, 35], Tadpole [36], VelvetOptimiser [88]. A5-miseq did not apply to Ion Torrent data.

To compare assembler performance, we reduced all data sets to 80x coverage. Total length, GC content, assembly contiguity (N50, NA50, and NGA50), error rate, genomic fraction, genomic features (complete + partial), the complete Busco, and the errors/per 100 kb were the chosen metrics for the evaluation of the assemblers (Supplementary Table 2) [50]. Optimal results were defined as maximal contiguity with minor errors.

All assemblers were analyzed for contiguity, with the contig sizes in terms of NA50. Except Tadpole with a very low N50 of 6.55 and MaSuRCA with a relatively high error rate (25.5 errors per 100 kb), all assemblers were able to produce assemblies with an N50 value between 20 and 30 kb with at most 130 contigs and at least 93% of genome covered using HiSeq data (Supplementary Fig. 3, 5 and 6). Assemblies based on MiSeq data were mainly unsatisfactory, with N50 values smaller than 20 kb. Notable exceptions were the assemblies produced by A5-miseq with N50 values of at least 27 kb and low error rates. N50 values of MIRA assemblies using Ion Torrent data were higher than those of any other assembler (26–43 kb), but due to large misassemblies, NA50 values were lower compared to SPAdes (Supplementary Fig. 4).

Subsequently, the genome fraction, the total number of aligned bases in the reference, is calculated and divided by the genome size [43]. Illumina HiSeq reads yielded the best results, while Ion Torrent data and MiSeq data resulted in a lower genome fraction (Supplementary Fig. 5). Here, ABySS and A5-miseq with Illumina HiSeq/MiSeq data performed best, whereby the latter method was capable of dealing with the poor sequencing quality of MiSeq reads analyzed before. MaSuRCAand SGA assemblies covered only a part of the genome with MiSeq and Ion Torrent data  (50% and 78%) (Supplemental Table 2).

**Fig. 3:**
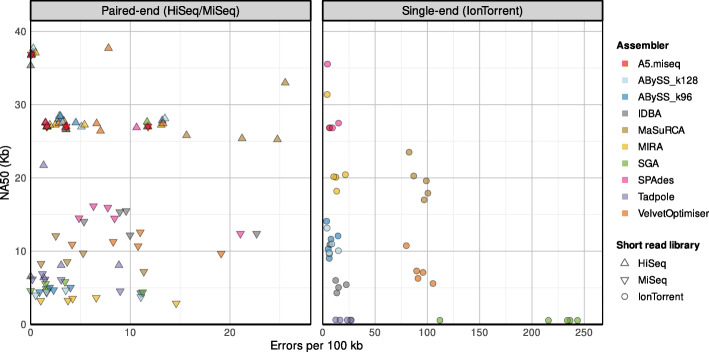
NA50 values versus assembly errors for short-read assemblies. Optimal results are expected to have high NA50 with low error rates (located in the upper left corner)

Additionally, a contig weighted score was calculated to represent an N50 value normalized to the contig number (Supplementary Fig. 7). Then we plotted the genome fraction versus the errors to combine an assessment of sequencing technologies and assembler performance (Fig. 3). For all assemblies, canSNPer yielded the same results as qPCR and short-read assemblies and are thus applicable in a diagnostic setup.

In conclusion, best assembly results were obtained for HiSeq data with Abyss_128k, for MiSeq data with A5-miseq, and Ion Torrent data with Mira. SPAdes yielded promising results, but performance is not optimal for the transposase library preparation used for MiSeq data. Assembly quality was assessed with Quast [43]. The duplication of the ~ 27 kb FPI could not be resolved in short-read assemblies.

### Analyzing assemblies of the reference strain

As a high-quality complete genome of *F. tularensis* subsp*. tularensis* strain SCHU S4 sequenced with Sanger is available with ASM898v1 we were able to make an in-depth evaluation of assembly quality using data from various sequencing platforms. To apply uniform conditions for comparison, we used randomly subsampled reads to obtain an average coverage of 50x for PacBio and MinION data and 80x for HiSeq, MiSeq, and Ion Torrent data (Table 2 and Table 3). Long-read assembly with Canu and Flye followed by polishing with Pilon resulted in assemblies with the highest contiguity concerning NGA50 values between 1.53 and 1.89 Mb. PacBio-based hybrid assemblies from Canu/Pilon were the best compared to all PacBio/ HiSeq assemblies of FSC237 isolate for contiguity with a length of 1.889.842 bp (compared to 1.892.775 bp in NC_006570.2), 99.85% of bases aligned to NC_006570.2 and 3794 covered genomic features. Although assemblies with Canu/Pilon had the highest NGA50 values, only 2 of 6 assemblies could be circularized properly with Circulator (Table 2 and 3).

**Table 2:** Hybrid assembly results for FSC237 isolate based on PacBio data

Assembler	Short read library	Total length (bp)	GC (%)	Contigs (> = 500 bp)	NGA50 (Mb)	Genome covered (%)	Genomic features (Complete + partial)	Complete Busco (%)	True/all circular contigs (size)	Errors (per 100 kb)
Canu/ Pilon	HiSeq	1,889,842	32.27	1	1.89	99.85	3794 + 3 part	93.92	0/0	4.34
	MiSeq	1,889,606	32.27	1	1.89	99.85	3794 + 3 part	92.57	0/0	17.14
	Ion Torrent	1,889,812	32.27	1	1.89	99.85	3794 + 3 part	93.24	0/0	5.82
Flye/Pilon	HiSeq	1,892,761	32.26	2	1.50	100.00	3795 + 2 part	93.92	1/1 (1892709)	0.74
	MiSeq	1,958,505	32.22	2	1.53	100.00	3796 + 1 part	92.57	1/1 (1892639)	23.78
	Ion Torrent	1,958,909	32.22	2	1.53	100,00	3796 + 1 part	93.92	0/1 (393321)	28.16
SPAdes	HiSeq	1,858,769	32.28	2	1.12	98.20	3736 + 3 part	93.92	0/1 (1499404)	0.16
	MiSeq	1,830,140	32.33	29	0.09	96.69	3600 + 15 part	93.92	0/0	14.09
	Ion Torrent	1,858,056	32.28	2	1.50	98.17	3738 + 1 part	92.57	1/1 (1892668)	4.36
Unicycler	HiSeq	1,856,294	32.29	6	1.46	97.79	3703 + 7 part	93.92	1/1 (1892695)	0.05
	MiSeq	1,865,687	32.28	7	1.15	98.08	3730 + 4 part	93.92	0/1 (393314)	10.28
	Ion Torrent	1,855,936	32.29	6	1.46	98.06	3718 + 5 part	93.92	1/1 (1892586)	6.04

**Table 3:** Hybrid assembly results for FSC237 isolate based on MinION data

Assembler	Short read library	Total length (bp)	GC (%)	Contigs (> = 500 bp)	NGA50 (Mb)	Genome covered (%)	Genomic features (Complete + partial)	Complete Busco (%)	True/all circular contigs (size)	Errors (per 100 kb)
Canu/ Pilon	HiSeq	1,949,612	32.26	1	1.95	99.97	3794 + 3 part	93.24	1/1 (1891217)	53.90
	MiSeq	1,942,673	32.34	1	1.94	99.97	3794 + 3 part	62.16	0/0	351.18
	Ion Torrent	1,948,850	32.27	1	1.95	99.97	3794 + 3 part	88.51	1/1 (1890592)	78.43
Flye/Pilon	HiSeq	1,921,160	31.96	3	1.89	99.97	3793 + 3 part	77.70	1/1 (1893441)	98.56
	MiSeq	1,944,710	31.58	3	1.92	99.97	3792 + 3 part	64.86	1/1 (1913901)	1059.83
	Ion Torrent	1,921,860	31.95	3	1.89	99.97	3792 + 3 part	67.57	0/0	162.61
SPAdes	HiSeq	1,892,530	32.28	1	1.86	98.21	3740 + 2 part	93.92	1/1 (1891993)	0.16
	MiSeq	1,827,899	32.33	32	0.09	96.58	3592 + 15 part	93.92	0/0	20.84
	Ion Torrent	1,858,435	32.28	2	1.40	98.19	3736 + 2 part	91.89	0/1 (1498148)	4.57
Unicycler	HiSeq	1,892,775	32.26	1	1.89	100.00	3794 + 3 part	93.92	0/0	0.05
	MiSeq	1,921,618	32.25	12	1.89	99.88	3789 + 3 part	93.92	1/1 (1891153)	57.40
	Ion Torrent	1,892,630	32.26	1	1.89	100.00	3794 + 3 part	93.92	0/0	6.77

In contrast, 4 out of 6 Flye/Pilon assemblies could be circularized appropriately. The covered genome fraction of the reference exceeded 97% in all assemblies except for those assembled with SPAdes based on MiSeq data. Most genomic features were found in Canu/Pilon and Flye/Pilon assemblies with at most 3796 complete and one partial. Very few assembly errors occurred in SPAdes and Unicycler assemblies, with only 0.05 errors per 100 kb for Unicycler assemblies based on HiSeq data. Conversely, Flye showed the highest number of errors with about 1060 errors per 100 kb using MinION and MiSeq data. SPAdes often produced large misassemblies caused by an extensive repeat, the FPI, whereas Unicycler resulted in a misassembly only with PacBio/MiSeq data (Supplementary Table 4, Fig. 5). A high number of misassembled bases identified by QUAST were detected around the origin of replication. This was correlated to the overall error rate, which is highest with MiSeq data, concordant with the GC bias.

Further evaluation of assembly errors in FSC237 with DNAdiff showed more GIndels in Canu/Pilon assemblies compared to others for PacBio/HiSeq and PacBio/MiSeq data (Supplementary Fig. 11). Flye/Pilon showed the most GIndel errors in all MinION assemblies (Supplemental Table 4). Analysis of assemblies with REAPR revealed twice as many errors in Flye/Pilon assemblies using PacBio/MiSeq data and  nearly three times more errors using PacBio/Ion Torrent data  compared to Canu/Pilon. With MinION data, the number of REAPR errors is similar in Canu/Pilon and Flye/Pilon assemblies. As noted before Unicycler and SPAdes assemblies had the lowest number of errors. Nevertheless, unlike QUAST results, Unicycler assembly had one third more GIndels for PacBio/Ion Torrent data and more than four times more GIndels for MinION/MiSeq data than SPAdes. The occurrence of FPI at two positions in the genome poses a central problem to assemblers, as seen in assembly graphs (Supplementary Fig. 17).

### Evaluation of hybrid assembly methods

We computed hybrid assemblies for each combination of long reads (PacBio, ONT) and short reads (HiSeq, MiSeq, Ion Torrent) using Canu/Pilon, Flye/Pilon, SPAdes, and Unicycler.

We compared NGA50, genome fraction, and errors per 100 kb aligned sequence in assemblies of five *F. tularensis* isolates based on their respective reference genomes. As before, we used subsampled data for the analysis. In general, the “long-read first” approaches (Canu/Pilon and Flye/Pilon) were more prone to sequence errors (mismatches, Indels) compared to “short-read first” approaches (SPAdes and Unicycler). Canu/Pilon assemblies based on PacBio data had often the highest NGA50 values among all results, with 9 out of 15 above 1.5 Mb (Supplementary Fig. 8, Supplementary Table 3).

In contrast, Flye/Pilon resulted in the best assemblies for MinION data with 9 out of 15 above 1.5 Mb. Canu/Pilon and Unicycler had the lowest overall failure rate with only 3 out of all 30 assemblies with NGA50 values below 0.5 Mb. Unicycler assemblies occasionally had lower NGA50 values compared to Canu and Flye. All hybrid SPAdes assemblies based on MiSeq reads showed very low NGA50 values smaller than 0.1 Mb. Coverage of reference genomes was higher with PacBio than MinION data with values above 98% in Canu and Flye assemblies (Supplementary Fig. 9). Flye assemblies had a higher genome fraction compared to Canu assemblies with MinION data. Hybrid assembly with Unicycler and HiSeq reads resulted in the smallest assembly errors, including mismatches and Indels (Supplementary Table 4). Looking at cumulated error rates in assemblies based on PacBio data, the results were similar for all methods ranging from 0.01 to 40.47 errors per 100 kb, where Flye had slightly more errors altogether (Supplemental Fig. 10, Supplemental Table 4). Long read assembly resulted in a much higher error rate with MinION data compared to hybrid methods. The highest error rate was observed in Flye/Pilon assemblies with more than 1000 errors per kb. Assembly polishing with Pilon was not very successful with MiSeq reads as the highest error rates occurred in those assemblies (Supplementary Fig. 10). In general, hybrid assemblies that used MiSeq data generated with the transposase library resulted in more misassemblies, mismatches, and Indels compared to those that used HiSeq or Ion Torrent data. SPAdes assemblies had the lowest overall error rate but were considerably more often affected by large misassemblies (Supplemental Table 4). Assemblies with the lowest error rate were produced by hybrid assembly with Unicycler based on PacBio/HiSeq or MinION/HiSeq data.

**Fig. 4:**
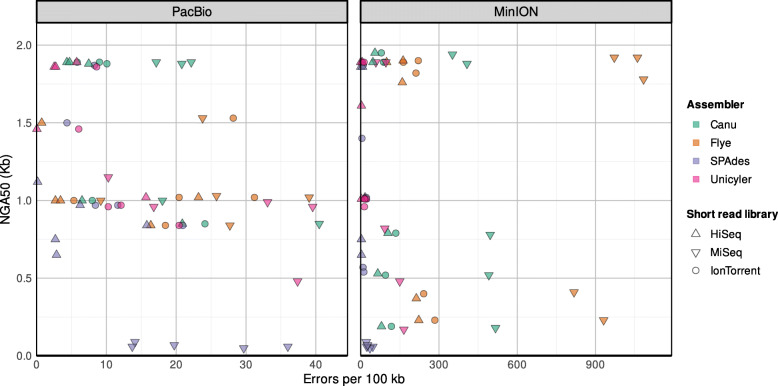
NGA50 values versus assembly errors for hybrid assemblies

**Fig. 5:**
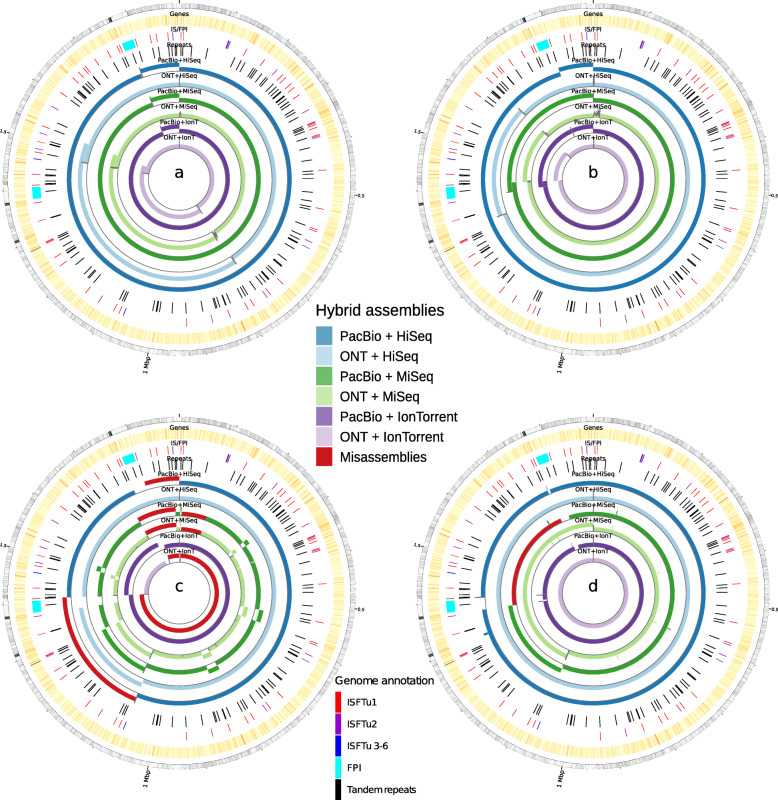
Genome assemblies of FSC237 isolate based on different sequencing platforms and assemblers aligned to the SCHU S4 reference genome **a**) Canu **b**) Flye **c**) SPAdes and **d**) Unicycler. Assembled contigs from inside to outside: ONT+Ion Torrent, PacBio+Ion Torrent, ONT+MiSeq, PacBio+MiSeq, ONT+HiSeq, PacBio+HiSeq;  misassembled bases identified by QUAST (dark red); mismatches in total error (grey bars); outer circle: *F. tularensis* subsp. *tularensis* str. SCHU S4 reference, inner circle: *F. tularensis* subsp. *tularensis* str. SCHU S4 RefSeq genes (yellow) and pathogenicity islands (cyan bars) and ISFTu 1-6 (red/ purple/ blue bars), repeats (black) presented with Circos. The origin of replication is at twelve o’clocd

ONT-based hybrid assemblies are more error-prone than PacBio-based hybrid assemblies. For SPAdes and Unicycler assemblies with Ion Torrent and MiSeq reads misassemblies profoundly impacted the assembly structure (Fig. 5). Canu/Pilon avoided large misassemblies with ONT reads, possibly due to its inbuilt read-error correction by computation of consensus reads.

The variability of assembly quality was higher with ONT data than PacBio data correlating with the read length and read quality (Supplemental Table 1).

In summary, we evaluated the NGA50 values versus the errors (Fig. 4) to combine an assessment of sequencing technologies and genome fidelity. PacBio sequences resulted overall in a better genome fidelity and lower errors. In terms of both contiguity (NGA50) and error rate, optimal assemblies were produced by Canu/Pilon and Unicycler. Flye/Pilon assemblies could be circularized more often than those from the other methods e.g. for subsampled data in 9 cases in contrast to 7 in Unicycler, 5 in Canu/Pilon, and 2 out of 30 in SPAdes (Supplementary Table 5).

### Genomic variants in *holarctica* isolates

To assess the application of all significant short-read sequencing technologies in diagnostics, we conducted a variant analysis.. We computed SNPs and Indels using short reads from all platforms. HiSeq reads had the lowest sequencing error rate and were sequenced to extremely high coverage of more than 1400x. We used them to determine the actual variation between our isolates and their reference genomes (Table 4). As expected, no difference in SNPs and Indels was found between FSC237 compared to the SCHU S4 reference. We found 35–207 SNPs and up to 2 Indels in *F. tularensis subsp. holarctica* isolates relating to their reference. Accordingly, 11 mismatches per 100 kb are actual variations in isolate 08 T013, but only 5 and 2 per 100 kb can be expected in the isolates 12 T0058 and 12 T0050/ 12 T0052. SNP calling was more robust with all sequencing technologies, while Ion Torrent  data were prone to additional Indel calling.

**Table 4:** Genomic variants in holarctica isolates with respect to their reference genomes called with three different short-read sequencing datasets

	Isolate	FSC237	08 T013	12 T0050	12 T0052	12 T0058
	NCBI ID	NC_006570.2	NC_017463.1	NC_009749.1	NC_009749.1	NC_019551.1
HiSeq	Average coverage	3910x	4793x	1432x	3232x	7711x
	SNPs	0	207	35	35	96
	Indels	0	2	0	0	0
Miseq	Average coverage	140x	102x	79x	78x	89x
	SNPs	81	312	153	173	246
	Indels	0	67	9	12	11
Ion Torrent	Average coverage	185x	204x	202x	163x	92x
	SNPs	0	189	49	47	94
	Indels	1	619	611	617	924

### Effect of coverage on assemblies

We determined the required coverage of both long and short reads for SPAdes and Flye/Pilon to achieve assemblies from FSC237 isolate of sufficient quality (Supplemental Fig. 12 and 13). SPAdes produced assemblies with an N50 value of 1.6 Mb with 10x coverage of long reads from Pacbio combined with 90x coverage of short reads from HiSeq. A higher long read coverage of 50x and short read coverage of 20x produced lower N50 values of about 1.5 Mb. SPAdes required far fewer Ion Torrent reads to produce good assemblies with an N50 value of 1.5–1.9 (minimum 20x) combined with long PacBio reads of at least 20x coverage. N50 values of SPAdes assemblies were very low with MiSeq data with a maximum of 0.16 Mb for 20x long read coverage and 140x short read coverage. SPAdes assemblies based on long reads from ONT data resulted in high variability of N50 values depending on the selected read subset. Flye required 30x coverage for PacBio and 20x for MinION reads to produce assemblies with an N50 value of at least 1.5 Mb. Covered genome fraction was at least 97% for all SPAdes assemblies except those using MiSeq data with a minimum of 85%. Flye required at least 20x coverage for PacBio and MinION to cover 98% of the reference genome. Assembly errors such as mismatches, Indels, and misassemblies might impair assembly quality. SPAdes assemblies based on HiSeq reads of at least 20x coverage had an error rate of less than one error per 100 kb (Supplemental Fig. 12). Flye/Pilon assemblies resulted in more errors with PacBio/HiSeq data with 0.1–1.9 errors per 100 kb for a PacBio coverage of 533x (Supplemental Fig. 13). They showed a lower error rate with PacBio/MiSeq and PacBio/Ion Torrent data than those produced with SPAdes using all PacBio reads (533x), but a much higher error rate with MinION with more than 100 errors per 100 kb independent of the coverage.

### Effects of preprocessing on assemblies

Down-sampling, error correction and filtering reads (to reduce misassemblies due to repeats) might benefit the assembly quality [24, 37, 46, 51]. We compared subsampling (50x), error correction with Canu and filtering of reads that reach a minimum length of 10 kb. Subsampling and error correction of PacBio reads improved NGA50 values of some Canu/Pilon assemblies, but with a reduced genome fraction (Supplemental Fig. 14, Supplemental Table 5). Conversely, preprocessing of MinION reads decreased NGA50 values and genome fraction in Canu/Pilon, Flye/Pilon and Unicycler assemblies. Error correction of PacBio reads raised the error rate in assemblies (Supplementary Fig. 15). Canu was also strongly affected by the filtering process, which lowered the NGA50 of six assemblies to half of the actual genome size. Flye could maintain an NGA50 of one in five assemblies independent of preprocessing, although it resulted in worse quality. Filtering of PacBio reads with a minimum length of 10 kb  substantially increased error rates with Flye/Pilon. Error correction of MinION reads reduced total errors in Flye/Pilon assemblies (Supplementary Fig. 15).

In summary, preprocessing of MinION reads might affect assembly quality by increasing error rates and decreasing NGA50 values. With original MinION data, most assemblies have a high NGA50 value over 1.5 Mb and a relatively low error rate, thus can be found in the upper left corner in the graph provided in Supplemental Fig. 16. The error rate of some erroneous Flye/Pilon assemblies was improved with error correction. Subsampling reduced the maximum number of detected errors in all assemblies from about 64 errors to 40 with PacBio data and 1300 to 1083 with MinION data.

### Performance comparison

We calculated running time and maximal RAM consumption with subsampled data (Table 5). Canu/Pilon and Unicycler had the highest needs in time and RAM, while SPAdes could finish the assemblies with substantially less time and RAM with at most 6 min and the need for 2.7 Gb RAM. Flye version 2.5 had an improved performance requiring 67 min (Table 5). Also, RAM consumption was highest with MinION data and Canu or Flye assembly with up to 10 Gb.

**Table 5:** Maximum RAM consumption and running time for assembly of FSC237 isolate with subsampled data (PacBio: 94 Mb, MinION: 92 Mbp, HiSeq: 151 Mb)

Assembler	Max RAM (Gb)		Running time (min)	
	PacBio	MinION	PacBio	MinION
Canu 1.8	3.56	6.06	27.93	97.03
Flye 2.4.2	2.64	10.33	6.91	96.91
Flye 2.5	2.60	7.42	7.26	67.43
SPAdes 3.13.0	2.74	2.70	6.09	5.84
Unicycler 0.4.7	8.00	6.01	52.32	44.32

### Evaluation of pathogenomic regions

#### Francisella pathogenicity island

**Fig. 6:**
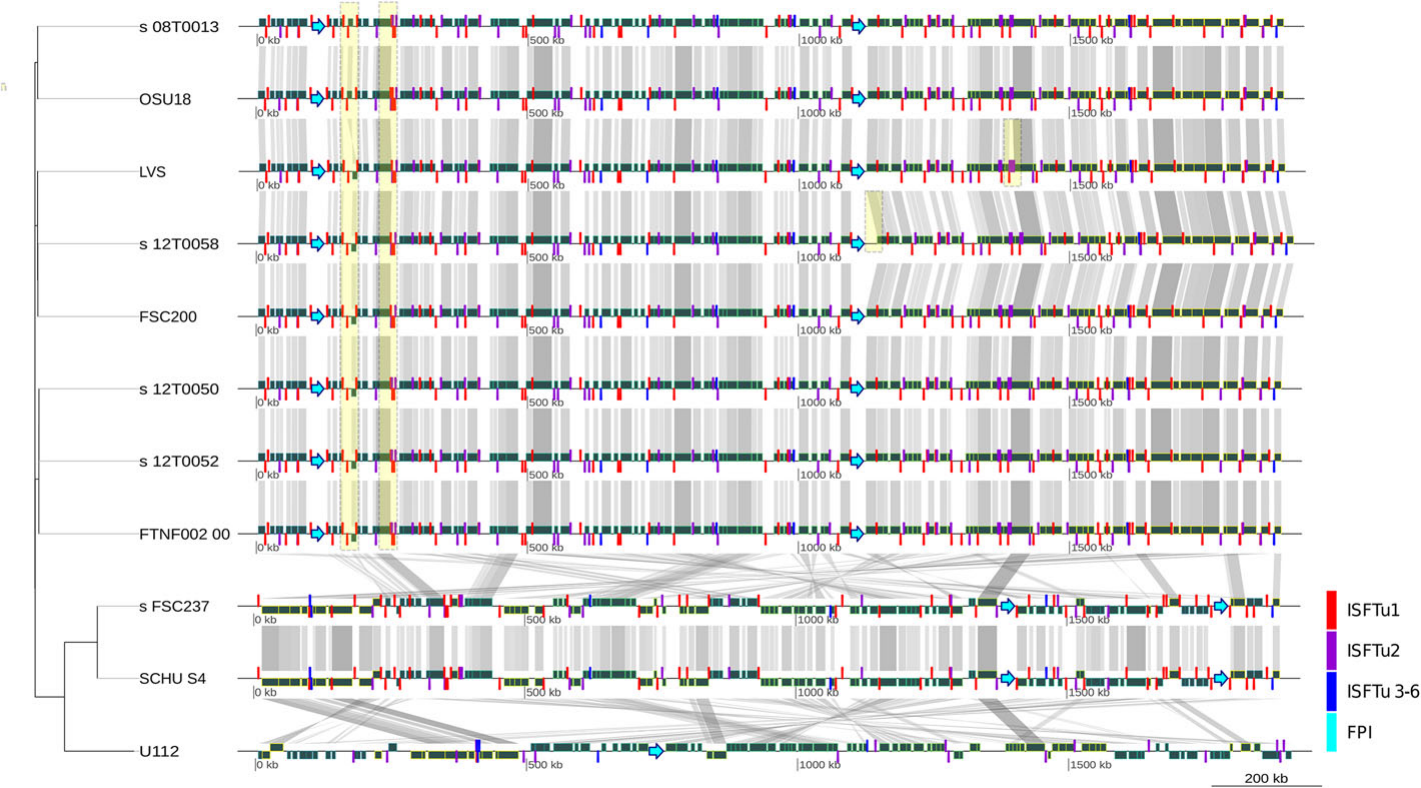
Alignment of genomes including Francisella pathogenicity island (FPI) and insertion sequences (IS or ISFtu) as coloured bars with neighbor joining tree based on kSNP on the left side

For hybrid assemblies, the comparison was made with downsampled sequences (50x long/ 80x short reads). The sequence alignment of the genomes revealed sequences for FPIs (Fig. 6). Misassembly of the FPI sequences disabled circularization or resulted in two chromosomal rings (Supplementary Fig. 17).

#### Host-vector genotyping

The heterogeneity of *Francisella* isolated from host and vector was analyzed comparing 12 T0050 from a hare and 12 T0052 from its sucking tick. Based on the remapping of HiSeq data, variations between 4 and 12 SNPs were found due to shortened repeats in the HiSeq data or wrong mapping. These SNPs could be excluded with manual curation. Thus, no difference between the isolates from the host and the vector could be detected. Additional SNP typing and phylogenetic analysis was done with Geneious comparing the genome 12 T0050 and 12 T0052. The result confirmed the absence of SNP variants between the two *F. tularensis* isolates from host and vector.

#### Erythromycin resistance

The erythromycin resistance of the isolates showed a perfect correlation with the phylogenetic group B.12 (Table 6). Only B.12 strains had an A-C SNP at position 2059 in the three copies of the *rrl* gene as reported before [52].

**Table 6:** Clades, genes, insertion sequences and FPI in isolates and reference strains. The number of detected insertion sequences in assembled genomes corresponds to those in respective references (Table 4)

	08 T0013	12 T0050	12 T0052	12 T0058	FSC237	NC_017463.1	NC_009749.1	NC_019551.1	NC_006570.2	NC_007880.1	NZ_CP009633.1
Strain	*F. Jul. holarctica* isolate	*F. Jul. holarctica* isolate	*F. Jul. holarctica* isolate	*F. Jul. holarctica* isolate	*F. Jul. tularensis* isolate	*F. Jul. holarctica* OSU18	*F. Jul. holarctica* FTNF002–00	*F. Jul. holarctica* FSC200	*F. Jul. tularensis* SCHU S4	*F. Jul. holarctica* LVS	*F. Jul. novicida* U112
Source	Hare, Ehingen (Bavaria, Germany) 2008	Hare, Herringhausen (North Rhine-Westphalia, Germany)	Tick, Germany	Hare, Heideck (Bavaria, Germany)	Human, Ohio 1941	Dead beaver found near Red Rock, Okla. 1978	Human, France 2002	Human, Ljusdal, Sweden 1998	Human, Ohio 1941	Live vaccine strain, Russia 1930	Turbid saltwater, Utah 1950
Clade	B.4	B.11	B.11	B.33	A.I.13	B.4	B.11	B.12	A.I.13	B.24	T/N.1
Size (bp)	1,893,712	1,890,831	1,890,859	1,913,616	1,892,771	1,895,727	1,890,909	1,894,157	1,892,775	1,895,994	1,910,592
Genes	2153	2151	2149	2164	2080	2162	2145	2143	2078	2147	1845
CDS	2104	2102	2100	2110	2031	2113	2096	2094	2029	2098	1796
rRNA	10	10	10	13	10	10	10	10	10	10	10
tRNA	38	38	38	40	38	38	38	38	38	38	38
tmRNA	1	1	1	1	1	1	1	1	1	1	1
FPI	2	2	2	2	2	2	2	2	2	2	1
ISFtu1	59	59	59	59	53	61	59	59	53	59	1
ISFtu2	42	42	42	42	16	42	42	42	16	43	17
ISFtu3	3	3	3	3	3	3	3	3	3	3	5
ISFtu4	2	2	2	2	1	2	2	2	1	2	1
ISFtu5	1	1	1	1	1	1	1	1	1	1	0
ISFtu6	1	1	1	1	1	1	1	1	1	1	0

#### Insertion sequences

The insertion sequences (IS or ISFtu) are short repetitive sequences that are evolutionary important and help to understand the evolutionary structure. Sequence alignment of the genomes revealed 123 ISFtu insertion sequences (Fig. 6, Table 6). ISFtu1 – ISFtu6 of FSC237 were assembled and annotated correctly at appropriate locations within the genome compared to the Sanger sequence.

The phylogenetic analysis allowed us to reveal the most ancient insertion sequence [53, 54]. Within all insertion sequences of the closest related species, a phylogenetic analysis was done. The insertion sequence with the name ISFtu1 CDS107 as part the *F. tularensis* ssp. *holarctica* 12 T0050 sequence was at the root of the phylogenetic tree. It can be suspected that this is the most ancient ISFtu. The differentiation of *F. tularensis* ssp. *tularensis* FSC237 occurred later.

These could be distinguished in three groups with affinity propagation clustering (Supplemental Fig. 18). The changed order of these insertion sequences or chromosomal segments suggests that regions moved during evolution. The chromosomal segments were nearly identical. However, they were differently arranged, and affinity propagation clustering revealed three distinct clusters independent of the previously annotated ISFtu1–6 describing a closer phylogenetic and evolutionary relation than suspected before.

## Discussion

### Data quality

To assess the sequencing data quality of all five sequencing technologies, we evaluated sequencing error, the coverage evenness, and the GC bias with mapping to the genome NC_006570.2. This strain was characterized by Sanger sequencing of the highest quality [55–59].

As expected, long sequencing reads were more error-prone as was reported before [26, 48, 60, 61]. In order to obtain the least possible insertion/ deletion rate, we suggest HiSeq reads for short-reads and PacBio for long-reads. The evenness score was very similar across all technologies. Although the same sequencing technology generated HiSeq and MiSeq results, bias could also be introduced by library construction and read length. This finding was congruent with other publications [62, 63]. While modest variation was seen for all sequencing technologies, the GC-bias observed for MiSeq data was striking. The transposases-dependent library preparation of Illumina Nextera XT and long-reading length appear to be causative. MiSeq reads can be improved by using stringent quality filtering, for example, with Trimmomatic or Sickle [64–67]. We suggest considering shorter reads (150 bp) with better quality values. Alternatively, we recommend cutting the reads to 150 bp and using a transposase independent library protocol to ameliorate the GC bias.

Long-reads of PacBio sequencing provided better data in terms of sequencing quality, error rate, and homogeneity. ONT sequencing is less expensive, with MinION as the only portable platform for potential field applications. Improved ONT technology and library preparation will probably result in better quality and longer reads in the future [61].

The assembly quality correlates with the sequencing quality, leading to the conclusion that errors in the sequencing data significantly impact assembly.

### Short-read assembly

Assembly programs have difficulties in properly locating reads from (almost) identical multicopy regions and induce fragmented assemblies, as was shown here due to the presence of two copies of the FPI. We benchmarked eight de novo assembly tools focused on short-read sequencing data. Each examined tool proved capable of assembling a *Francisella* genome over 90%. However, all assemblers evaluated in this study are under constant development, and therefore, our data can only be a snapshot of their performance under the described circumstances.

Data from transposase-generated libraries should be filtered (Fig.1). Keeping in mind the uniqueness of the data set and the requirements for diagnostics, we recommend using assemblers that are adapted optimally to the characteristics of the data: A5-miseq for MiSeq, Mira for Ion Torrent and ABySS for HiSeq data. However, the FPI duplication will not be resolved.

### Hybrid assembly

We benchmarked four state-of-the-art software solutions that are freely available and applicable to the here used down-sampled datasets. Although long-reads feature relatively high base-calling error rates, polishing the Canu/Pilon or the Flye/Pilon assembly graph using short-reads reduced the error rates by one order of magnitude. The higher error rate of ONT reads could not be fully compensated. PacBio sequencing resulted in better genomes. Preprocessing was not always beneficial.

The assemblers Flye/Pilon and SPAdes are substantially faster than Canu but resulted in more errors and misassemblies. For datasets of eukaryotes, it was shown that Flye/Pilon provide more contiguous and accurate assemblies than Canu while being notably faster. However, our study with bacteria Canu/Pilon provided fewer errors [48]. The software Canu/Pilon and Unicycler had the best results concerning error rate and contiguity in our hands. Financial aspects, correctness, and availability of computational powers are often a concern, but establishing corrective measures is also relevant.

Overall, the “long-read first” approach of Canu/Pilon provided the best assembly by showing the correct structure of the genome with the least error rate.

### Pathogenomics

Accurate hybrid genome assembly is hampered by repetitive regions [68]. Although long sequencing reads are more able to resolve genomic repeats than short-read data, most long-read assembly algorithms do not provide the repeat characterization necessary for producing optimal assemblies.

The hybrid assemblies generated here led to striking misassemblies around the pathogenicity islands, shown with alignments to the reference. The ~27 kb duplication was the cause of major local misassemblies. The “short-read first” approaches (SPAdes and Unicycler) had a low total error rate but were prone to misassemble this duplication. The “long-read first” approach Canu/Pilon provided the correct assembly of this duplication. Misassembly could, however, be prevented with higher coverage.

The assessment of the genomes obtained from a host (12 T0050) and vector (12 T0052) showed no genetic differences, which was expected from previous reports [52, 69]. The detected antibiotic resistance of clade B.12 to erythromycin was in agreement with previously reported results [52]. The heterogeneity within the bacterial genomes of strains isolated from a host and its vector was so minimal that chromosomal variations could not be detected due to the assembly artifacts mentioned above.

Insertion sequences are transposable elements capable of increasing their copy number within the genome, and they can move within the genome. In the genomes of pathogenic *F. tularensis* strains, the most common insertion sequences are ISFtu 1 (IS630) and ISFtu2 (IS5), which belong to the Class II of mobile elements. They transpose by excision, and the subsequent reinsertion occurs at random genomic loci [5, 6, 70, 71]. ISFtu1 belongs to the Tc-1 mariner family of insertion sequences and has a single open reading frame [72, 73]. ISFtu1 is highly regulated, as was shown previously in published genome structure analyses [74, 75]. Ribosomal frameshifting is required to translate ISFtu1 as the DDE triad, which is essential for transition at the IS positions, which is generated only after a frameshift. The ribosomal frameshifting motif may be used to control the transposition of insertion sequences. The analysis with affinity propagation clustering suggests that this happened three times in FSC237. Insertion sequences in the same order suggest that these were existent before the human pathogenic subspecies differentiated from a common ancestor. The other insertion sequences that differ in content and order can be presumed to have formed after differentiation. We concluded that the subspecies and clades split early.

*Normally, a phylogenetic tree* shows the relatedness of species when based on rRNA or whole genome sequences. Here we used a *phylogenetic tree* to show the relatedness between the ISFtu. The difference to the traditional rRNA sequences tree is explained that does not base on the most common ancestor under a continuous mutation rate, but on the actively jumping ISFtu. As reported before the observed genome to genome variation in gene content and IS elements were different and reinforces the view that similar evolutionary paths of host adaptation might have developed independently [76].

Insertion sequences with the highest identity (70%) to *F. tularensis* can be found in the pathogens *Piscirickettsia salmonis, Orientia tsutsugamushi,* and *Legionella pneumophila* (blastn [77], indicating a general mechanism with which insertion sequences can give rise to pathogenicity or adaption to the hosts or environment as known from other species [53, 54]*.*

The results are shown here (Fig. 5) indicate that all assemblers provided high error rates around the origin of replication. The structure of the origin of replication is AT-rich and repeat-rich and might thus complicate the assembly.

Better algorithms for resolving repeats in assembly graphs might have the potential to improve bacterial assemblies significantly by increasing their contiguity and reducing the error rate. Assembly graphs as generated with SPAdes, Flye/Pilon, and Bandage can also be used to create breakpoint graphs [44] and are helpful tools to analyze structural variations.

## Conclusions

Our study evaluated five sequencing technologies to assess the genome of *F. tularensis*. Our data show that short-reads are less error-prone than long-reads. HiSeq and PacBio provided the best results among the respective technologies (and in combination for creating hybrid genome assemblies). The sequencing quality corresponded to assembly quality. Short-read sequencers provide high-quality data suitable for genotyping and diagnostics. Except for the MinION platform, they are generally cheaper per base pair compared to long-read sequencers.

For the assembly of Francisella with HiSeq data, Abyss_128k proved optimal. However, alternative combinations such as MiSeq data and A5-miseq are acceptable, while Mira generated with Ion Torrent data eligible assemblies in our study.

Hybrid assembly strategies were assessed to establish optimal genome assemblies. The “long-read first- error correct - with short-reads afterward” approach as performed with Canu/Pilon resulted in the best results.

The duplicate FPI is essential for the host-pathogen interaction and has to be resolved correctly, but it could be a significant cause for misassembly. The detailed genomes allowed an evolutionary analysis of insertion sequences, revealing a highly regulated adaption process of *Francisella*. Other bacteria that have similar genome structures as *Francisella* might be analyzed with our strategy.

## Methods

### Strain selection and reference genomes

Four *F. tularensis* subsp*. holarctica* strains 08 T0013, 12 T0050, 12 T0052, 12 T0058, and *F. tularensis* subsp*. tularensis* FSC237 were used in the present study. All *F. tularensis* subsp*. holarctica* strains were isolated on cysteine heart agar from carcasses of hares (*Lepus europaeus*), the main source of infection in Germany [78]. The strains were assigned to the subclades using a set of real-time PCR assays and bioinformatics analysis using CanSNPer (https://github.com/adrlar/CanSNPer), which is an assay for whole-genome sequencing data based on canonical single nucleotide polymorphisms developed by Larkeryd et al., 2014 [79]. Strain 08 T0013 was isolated near Ehingen (Bavaria, Germany) in 2008. The subclade was identified as clade B.4. We used an isolate from OSU18 (NC_017463.1) as a reference. 12 T0050 was isolated in Herringhausen (North Rhine-Westphalia, Germany) and the clade was identified as B.6. The reference genomes were selected according to their reported subclade and were used as reference genomes for example in the canSNPer. For the strains 12 T0050 and 12 T0052, we used FTNF002–00 (NC_009749.1) as the reference genome. Strain 12 T0058 was isolated in Heideck (Bavaria, Germany), and the clade was identified as B.12. We selected FSC200 (NC_019551.1) as its reference genome. *F. tularensis* subsp. *tularensis* strain SCHU S4 (FSC237) (SCHU S4; NC_006570.2) was included as a control. Initially isolated in the US, FSC237 was obtained from the Institut für Mikrobiologie der Bundeswehr (Munich, Germany) on 30 Nov. 2006 in a cryotube. Viability was checked in 2007 and 2013, but the strain was not passaged in subsequent cultivations.

The DNA sequence of the FPIs was used from *F. tularensis* subsp. *novicida* strain U112, (GenBank accession no. AY293579) [19]. For the insertion sequences the following sequences were used as annotation reference: isftu1: NC_006570.2: 1683438..1684222, isftu1 transposase, Gene ID: 3192293; ISFtu2: NC_006570.2: 383702..384445 isftu2 transposase, Gene ID: 3192483; ISFtu3 (discontinued) = FTH_1009 pseudo, NC_008369.1: 992987–993,874, Gene ID: 4307123; ISFtu4 (discontinued) = pseudogene of ISFtu4 transposase, NC_008369.11610822..1611085, complement; ISFtu5 (discontinued) = FTH_0855 pseudo NC_008369.1 (637,547..637960, complement), Gene ID: 4306969; ISFtu6 (discontinued) = FTH_0855 pseudo NC_008369.1 (850,708..851016), Gene ID: 4306969 (Table 7).

**Table 7:** Strain selection and reference genomes

Species	Isolate	Clade	Reference strain (NCBI ID)	Reference assembly (RefSeq ID)	Reference genome size
*F. tularensis subsp. tularensis*	FSC237	AI	SCHU S4 NC_006570.2	GCF_000008985.1	1,892,775
*F. tularensis subsp. holarctica*	08 T0013	B.4	OSU 18 NC_017463.1	GCF_000011405.1	1,895,727
	12 T0050	B.6	FTNF002–00 NC_009749.1	GCF_000017785.1	1,890,909
	12 T0052	B.6			
	12 T0058	B.12	FSC 200 NC_019551.1	GCF_000168775.2	1,894,157

### DNA extraction and sequence generation

The cultivation of bacteria from organ specimens was performed on cysteine heart agar at 37 °C with 5% CO_2_ for 48 h. DNA for whole-genome sequencing was prepared from a 10 mL culture in brain heart infusion broth. Bacterial cells were harvested after 72 h by centrifugation, and the DNA was purified using QIAGEN Genomic-tip 20/G and a QIAGEN Genomic DNA buffer set kit (Qiagen, Hilden, Germany). The DNA quality was examined using a Qubit 2.0 fluorometer (Life Technologies, Germany) and agarose gel electrophoresis.

### Library preparation and sequencing

Illumina Nextera XT libraries were uniquely barcoded, pooled, and run on a MiSeq flow cell with paired 250 base reads plus an 8-base index read. According to the manufacturer’s instruction, one ng of the genomic DNA was prepared with the Nextera XT library preparation. The resulting libraries were sequenced in a 250 bp Illumina MiSeq paired-end sequencing run. HiSeq libraries were constructed by GATC (Konstanz, Germany) using TruSeq protocols and were sequenced on a single lane of an Illumina HiSeq with paired 75 base reads plus an 8-base index read. Ion Torrent libraries were used on single 316 chips with 65 cycles generating mean read lengths of 112–124 bases in each run. PacBio sequencing was performed with an amplification-free workflow. The genome sequencing was done with SMRT DNA sequencing [80] using a PacBio RSII sequencer. Standard PacBio libraries contained inserts with an average of 2 kb. The libraries were run individually over multiple SMRT cells using C1 chemistry. For each genome, ≥20x sequence coverage data was obtained (GATC, Konstanz, Germany).

Nanopore sequencing libraries were prepared with one μg genomic DNA using the 1d^2^ kit (SQK-LSK308). DNA was not sheared before library preparation and was end-repaired and dA-tailed. An individual R9.5 flow-cell was used for each sample, providing ≥30x sequence coverage data for each genome. The technical specifications of the platform were summarized in Table 1.

### Base-calling

Base-calling of ONT reads was performed with Albacore version 1.7.4 and version 2.0.2 (ONT). Initial quality control and data inspection were performed using NanoOK [81]. For ONT, Albacore was used for base-calling with standard parameters. For PacBio sequencing, HGAP algorithm version 3 (RS_HGAP_Assembly.3) implemented in PacBio SMRT portal version 2.3.0 was used [82].

### Quality assessment of raw reads

Quality statistics for raw fastq data were calculated using PRINSEQ-lite, version 0.20.4 [67]. Reads were aligned against the respective reference genome using BWA (version 0.7.17) and sorted by coordinate with Samtools (version 1.3.1). Long reads from PacBio and ONT were alternatively mapped with Minimap2 (version 2.16-r922). We used QualiMap (version 2.2.1) to approximate sequencing error rate and GC percentage with strain FSC237 as described below. Substitutions and Indels relative to the reference genome were computed from CIGAR values of mapping results. The general error rate was calculated as the total collected edit distance ratio to the number of mapped bases in percent. GC percentage of reads was computed from all alignments. Per-scaffold average coverage and GC percentage of the reference genomes were calculated using the script *pileup.sh* from BBMap (version 38.22).

To analyze the distribution of coverage across protein-coding genes for each sequencing platform, we used the bedtools package [83] and custom scripts written in Python and R. We converted BAM files to bedGraph format and intersected the bedGraph file with CDS regions from the RefSeq genome annotation (as suggested by Barbitoff [84]. We calculated coverage evenness score E across genes as described in Mokry et al., 2010 [85].

We evaluated the GC bias of each platform from sorted alignments using *CollectGcBiasMetrics* from Picard tools (version 2.14.0), which computes a relative measure of sequence coverage by the reads with a certain GC content. We used local polynomial regression fitting (loess) with ggplot2 to plot the distribution of GC versus normalized coverage.

### Subsampling of reads

For better comparability of sequencing platforms, sequencing data were subsampled from FSC237 to determine the minimum read depth required for complete assemblies. Reads from MiSeq, HiSeq, Ion Torrent, PacBio, and ONT sequencers were randomly subsampled using Seqtk to achieve a target coverage between 10x and 100x to the reference genome calculated using the mean sequence read length.

### Curation of the final genome assembly

Based on the Sanger sequence as a reference genome, assemblies were adjusted manually, applying Contiguator [86] and Circulator [87] to obtain the final assemblies. Remapping was done for all final genomes.

### Short-read assembly

Paired and single-end reads from HiSeq, MiSeq, and Ion Torrent were subsampled to a target coverage of 80x to the reference genome. Paired-end reads with coverage of 80x from HiSeq and MiSeq were preprocessed using Trimmomatic (version 0.39) [66]. Gentle quality trimming and adapter clipping was applied (parameters ILLUMINACLIP:TruSeq3-PE.fa:2:30:10:2:keepBothReads LEADING:3 TRAILING:3 MINLEN:18 for HiSeq and ILLUMINACLIP:NexteraPE-PE.fa:2:30:10:2:keep Both Reads LEADING:3 TRAILING:3 MINLEN:18 for MiSeq reads).

Paired-end reads were assembled using A5-miseq pipeline, ABySS with kmer length of 96 and 128, IDBA, MaSuRCA, MIRA, SGA, SPAdes, Tadpole, and VelvetOptimiser using parameters as given in Table 8. Ion Torrent reads were processed with all assemblers in single-end mode except for A5-miseq, which cannot be applied for single-end reads.

**Table 8:** Assembler software for short-read assembly

Assembler	Assembly method	Version	Release date	Parameter
ABySS [28]	Single k-mer De Bruijn graph	2.2.3	27/09/2019	-k 96 / -k 128
A5-miseq [29]	Automated pipeline including read cleaning, k-mer based error correction, assembly with IDBA and misassembly correction	20,160,825	25/08/2016	default
IDBA [30]	Accumulated De Bruijn graph with iteratively increased k-mer size	1.1.3	11/07/2016	--mink 20 --maxk 124
MaSuRCA [31]	DeBruijn graph and Overlap-Layout-Consensus (OLC)	3.3.4	13/09/2019	GRAPH_KMER_SIZE = auto cwgErrorRate = 0.25 CLOSE_GAPS = 1
MIRA [32]	‘High-quality alignments first’ contig building strategy with iterative removal of technology-specific errors	V5rc2	26/02/2019	Default
SGA [33]	String graph based on read pair overlaps (using FM index)	0.10.15	05/08/2016	-m 111 --min-branch-length 400
SPAdes [35]	Multi-kmer De Bruijn graph	3.13.0	16/10/2018	--cov-cutoff auto --careful
Tadpole [36]	Single k-mer-based assembly with read extension optimized for correctness	BBMap 35.85	16/08/2016	Default
VelvetOptimiser [88]	Single k-mer De Bruijn graph with optimised N50	2.2.6 Velvet: 1.2.10	03/08/2017 05/07/2018	-s 97 -e 127 -x 10

### Hybrid assembly

Long-reads from PacBio and ONT were preprocessed with three different approaches:

1. Subsampling: Reads were randomly subsampled using Seqtk to 50x target depth and achieved an average coverage of 30-40x of the reference genome.
2. Correction: Subsampled reads were corrected using Canu (1.8) with default options.
3. Filtering (10 kb): Long-reads were filtered using Filtlong (v.0.2.0) with a minimum read length of 10 kbp keeping only the best reads up to 60 Mbp in total.

All short-reads were subsampled to a target coverage of 80x. Paired-end reads were preprocessed with Trimmomatic, as mentioned. Hybrid assemblies with long-read first approaches were computed using Canu and Flye combined with assembly polishing with Pilon, for Canu *stopOnLowCoverage* parameter was set to 5 and *genome size* *= 1.9 m*. Flye (v. 2.4.2) was applied with default parameters and *-g = 1.9 m*. Short-reads were aligned to the assembly with BWA (v. 0.7.17) and indexed using Samtools (v. 1.9). Assemblies were polished with Pilon (v. 1.23) using mapped short-reads (with parameters “*fix-all*” and “*mindepth* 0.5”).

The short-read first approach was applied using SPAdes (v. 3.13.0) with the parameters *–careful* and *–cov-cutoff* auto and Unicycler (v. 0.4.7) with default parameters.

### Assembly comparison metrics

To evaluate the completeness and quality of genome assemblies, several metrics were used:

1. **Contiguity:** The N50 metric has been widely adopted as a measure of assembly contiguity. The length-weighted median contig size means that half of the entire assembly is contained in contigs of length with at least this value. As large-scale misassemblies might confound the result, the N50 value is often corrected by breaking contigs at misassembled sites. Thus obtained NA50 metrics might be normalized by actual genome length to enable comparisons among assemblies of genomes of different sizes resulting in the NGA50 metric. We computed N50, NA50, and NGA50 values using QUAST.
2. **Completeness:** The *F. tularensis* genome consists of one circularized contig of 1.89 Mbp, which is also true for the subspecies *tularensis* and *holarctica*. We utilized Circulator (v. 1.5.5) [87] with Canu (v. 1.4) and SPAdes (v. 13.3.0) to circularize assemblies using corrected reads obtained using Canu. We consider an assembly as complete if it can be circularized correctly. In our study, we postulated that a circularized contig is at least 1.8 Mbp long. We accessed the completeness of the gene set measured by the percent of BUSCO (Universal single-copy Ortholog) genes found in the assembly in a complete or partial form.
3. **General assembly metrics: **We measured total length, number of contigs with at least 500 bp length, GC content, percentage of reference genome covered, and number of genomic features (genes, transcripts, CDS) in the assembly based on an annotated list of gene positions in the reference genome using QUAST (v. 5.0.2) [43]. Genomic features correspond to all features (genes, transcripts, CDS) in the reference annotation found complete or partial in the assembly. Complete Busco is the percentage of complete BUSCO (Universal Single-Copy Ortholog) genes found in the assembly.
4. **Assembly accuracy:** The number of assembly errors, i.e., the number of misassemblies, local misassemblies, misassembled contig length, and mismatches, Indels, and Ns per 100 kbp were calculated to the reference using QUAST (v. 5.0.2). Misassemblies per 100 kbp were computed from the number of misassemblies in a 100 kb aligned sequence. Total errors per 100 kbp comprise misassemblies, mismatches, and Indels. Additionally, total assembly errors were estimated by mapping of paired-end Illumina HiSeq reads (200x coverage) to the assembly using REAPR (v. 1.0.18) [89]. High-quality SNPs and Insertions/ Deletions (programmed as bounded by 20 exact, base-pair matches on both sides) were computed to the reference genome using DNAdiff (MUMmer 3.23) [90]. Assembly graphs were inspected using Bandage [44].

### Running time/ RAM comparison

Performance tests of assemblers were run on a server with 32 cores (2x Intel Xeon CPU E5–2667 v2 Octa-Core) and 387 GB RAM using eight cores (Table 9).

**Table 9:** Assembler software for hybrid assembly

Assembler	Version	Method	Read error correction	Assembly polishing
Canu + Pilon	1.8/ 1.23	Long-read first/ Best overlap graph (BOG)	consensus of long-reads from overlapping reads	Pilon
Flye + Pilon	2.4.2/ 1.23	Long-read first/Repeat graph	None	Pilon
SPAdes	3.13.0	Short-read first/ de Bruijn graph	BayesHammer (Illumina); hammer (Ion Torrent)	MismatchCorrector (default: disabled)
Unicycler	0.4.7	Short-read first/ de Bruijn graph (SPAdes) and string graph of short-read contigs and long-reads (Minasm)	BayesHammer (Illumina)	Racon + Pilon

### Variant calling

Traditionally whole genome sequences of *Francisella* were analyzed with canSNPer [79] elucidating a detailed analysis suitable here, although other methods provided useful analysis methods, as described elsewhere [91]. Reads were mapped to the reference genome with BWA (0.7.17). Alignment files were sorted by position and indexed with Samtools (1.3.1). Variants (SNPs and short Indels) were called using the mpileup command of Samtools along with Bcftols.

### Genomic analyses

IS elements were identified using the geneious annotation and extraction [92]. The alignment was done with geneious alignment with “*global alignment-free end gaps*” and 65% similarity. The Geneious tree builder was used in Takamura-Nei in Neighbor-Joining Tree mode with no outgroup. The distance matrix was exported and subjected to affinity propagation [93, 94].

FPI and Insertion sequences were located in assemblies and reference genomes using Blast with sequence AY293579 and IS elements ISF 1–6 as query. Blast was run with default options for IS and *–qcov_hsp_perc 80* for FPI. A phylogenetic tree was computed using kSNP 3.0 with *–k 12*. Genomes were aligned using progressive alignment of Mauve [95 ] . A genomic map with IS positions was computed using the R package *genoPlotR* [96] with the Mauve alignment as a backbone filtering blocks smaller than 5 kb. Genomes are ordered corresponding to the neighbor-joining tree from kSNP.

## Supplementary Information

**Additional file 1: Supplementary Figure 1**. Histograms of average coverage with density of gene regions for used sequencer platforms in FSC237 isolate.

**Additional file 2: Supplementary Figure 2**. Bivariate plot of log-transformed read length against base call quality with hexagonal bins and marginal histograms (NanoPlot) of raw reads a) PacBio and b) ONT.

**Additional file 3: Supplementary Figure 3**. Boxplots of N50 values for short-read assemblers.

**Additional file 4: Supplementary Figure 4**. Boxplots of NA50 values for short-read assemblers.

**Additional file 5: Supplementary Figure 5**. Boxplots of genome fraction for all short-read assemblers.

**Additional file 6: Supplementary Figure 6**. Violin-Boxplots of assembly errors per 100 kb for all short-read assemblers.

**Additional file 7: Supplementary Figure 7**. Violin-Boxplots of contig weighted score for all short-read assemblers.

**Additional file 8: Supplementary Figure 8**. Violin-Boxplots of NGA50 values for hybrid assemblies of all isolates.

**Additional file 9: Supplementary Figure 9**. Violin-Boxplots of genome fraction with respect to reference genomes for hybrid assembly of all isolates.

**Additional file 10: Supplementary Fig. 10**. Violin-Boxplots of assembly errors per 100 kb for hybrid assembly of all isolates.

**Additional file 11: Supplementary Fig. 11**. Break down of assembly errors in assembly of  FSC237 in comparison to SCHU S4 reference (GSNPs, GIndels, REAPR errors ). The vertical axis indicates the total number of error in whole genome.

**Additional file 12: Supplementary Fig. 12**. Effects of coverage on NGA50 values and errors in SPAdes assemblies.

**Additional file 13: Supplementary Fig. 13**. Effects of coverage on NGA50 values and errrors in Flye/P ilon assemblies.

**Additional file 14: Supplementary Fig. 14**. Effects of preprocessing on NGA50 and genome fraction in hybrid assemblies.

**Additional file 15: Supplementary Fig. 15**. Effects of preprocessing on errors in hybrid assembl ies.

** Additional file 16: Supplementary Fig. 16**. NGA50 values versus assembly errors for vari ous   prepr ocessing methods  in hybrid assemblies.

** Additional file 17: Supplementary Fig. 17**. Sequence alignments (green) with Francisella pathogenicity islands (blue) revealed them as a major cause for misassembly. To prevent Blast from generating artifacts, the minimum alignment length parameter were set to > 40 kb in Bandage or FPI sequences were masked prior to Blast.

**Additional file 18: Supplementary Fig. 18**. Affinity propagation clustering of all the 123 insertion sequences of FSC237 revealed 3 cluster.

**Additional file 19: Supplementary Table 1**. Read statistic of raw data and preprocessed reads.

**Additional file 20: Supplementary Table 2**. Performance of tested eight short-read assemblers in five isolates of *F. tularensis.*

**Additional file 21: Supplementary Table 3**. Performance of long-read and hybrid assemblers in five isolates of *F. tularensis.*

**Additional file 22: Supplementary Table 4**. Error rates in hybrid assemblies compared to references.

**Additional file 23: Supplementary Table 5**. Statistical analysis of hybrid assemblies/ preprocessing effects.

## Data Availability

All data generated and analyzed during this current study are available at the Friedrich-Loeffler –Institute, IBIZ, Jena, with permission from the Competent Authority. The Whole-genome sequence was submitted in NCBI Database having BioProject ID PRJNA625652.

## Abbreviations

bp: Base pairs
BUSCO: Universal Single-Copy Ortholog
CDS: Coding sequence
DDE: A protein domain containing an acidic amino acid triad (DDE or DDD) that catalyzes the “cut and paste” transposition reaction
DNAdiff: DNA difference = high-confidence SNPs and Indels of the assembly/ high-confidence SNPs and Indels of the reference genome
FPI: Francisella pathogenicity island
Gb: Gigabases
GIndels: Genomic Insertion and Deletion
GSNP: Genomic single nucleotide polymorphism
Indels: Insertions and Deletions
IS, or ISFtu: Insertion sequences or insertion sequences of *F. tularensis*
Kbp: Thousand base pairs
L50: L50 is the number of contigs whose summed length is N50
Mbp: Million base pairs
N50: The minimum contig length needed to cover 50% of the genome
NA50: NA50 (corrected NG50) correspondent to the assembly with the highest N50 results
NG50: Mean or median of lengths of contigs
NGA50: Defined as NG50, where the lengths of aligned blocks are counted instead of scaffold lengths
ONT: Oxford Nanopore Technologies
PacBio: Pacific Biosciences
QC: Quality control
RAM: Random-Access Memory
SNP: Single nucleotide polymorphism
