# Correction to: Testing assembly strategies of *Francisella tularensis* genomes to infer an evolutionary conservation analysis of genomic structures

**DOI:** 10.1186/s12864-021-08208-7

**Published:** 2021-12-14

**Authors:** Kerstin Neubert, Eric Zuchantke, Robert Maximilian Leidenfrost, Röbbe Wünschiers, Josephine Grützke, Burkhard Malorny, Holger Brendebach, Sascha Al Dahouk, Timo Homeier, Helmut Hotzel, Knut Reinert, Herbert Tomaso, Anne Busch

**Affiliations:** 1 Department of Mathematics and Computer Science, Algorithmic Bioinformatics, Freie Universität Berlin, Institute of Computer Science, Takustr. 9, 14195 Berlin, Germany; 2 German Federal Institute for Risk Assessment, Diedersdorfer Weg 1, 12277 Berlin, Germany; 3 Friedrich-Loeffler-Institut, Institute of Bacterial Infections and Zoonoses, Naumburger Str. 96a, 07749 Jena, Germany; 4 Department of Biotechnology and Chemistry, Mittweida University of Applied Sciences, Technikumplatz 17a, 09648 Mittweida, Germany; 5 Friedrich-Loeffler-Institut, Institute of Epidemiology, Südufer, 1017493 Greifswald, Insel Riems Germany; 6 Department of Anaesthesiology and Intensive Care Medicine, University Hospital Jena, Jena, Germany

**Correction to: BMC Genomics 22, 822 (2021)**

**https://doi.org/10.1186/s12864-021-08115-x**

Following publication of the original article [1], it was reported that Röbbe Wünschiers’ name was misspelled as Roebbe Wuenschiers.

The original article [1] has been corrected.
