# Supplementary figures and images for: Testing assembly strategies of Francisella tularensis genomes to infer an evolutionary conservation analysis of genomic structures
Source: BMC Genomics. 2021 Nov 14;22:822. doi: 10.1186/s12864-021-08115-x (PMC8590783; doi:10.1186/s12864-021-08115-x)

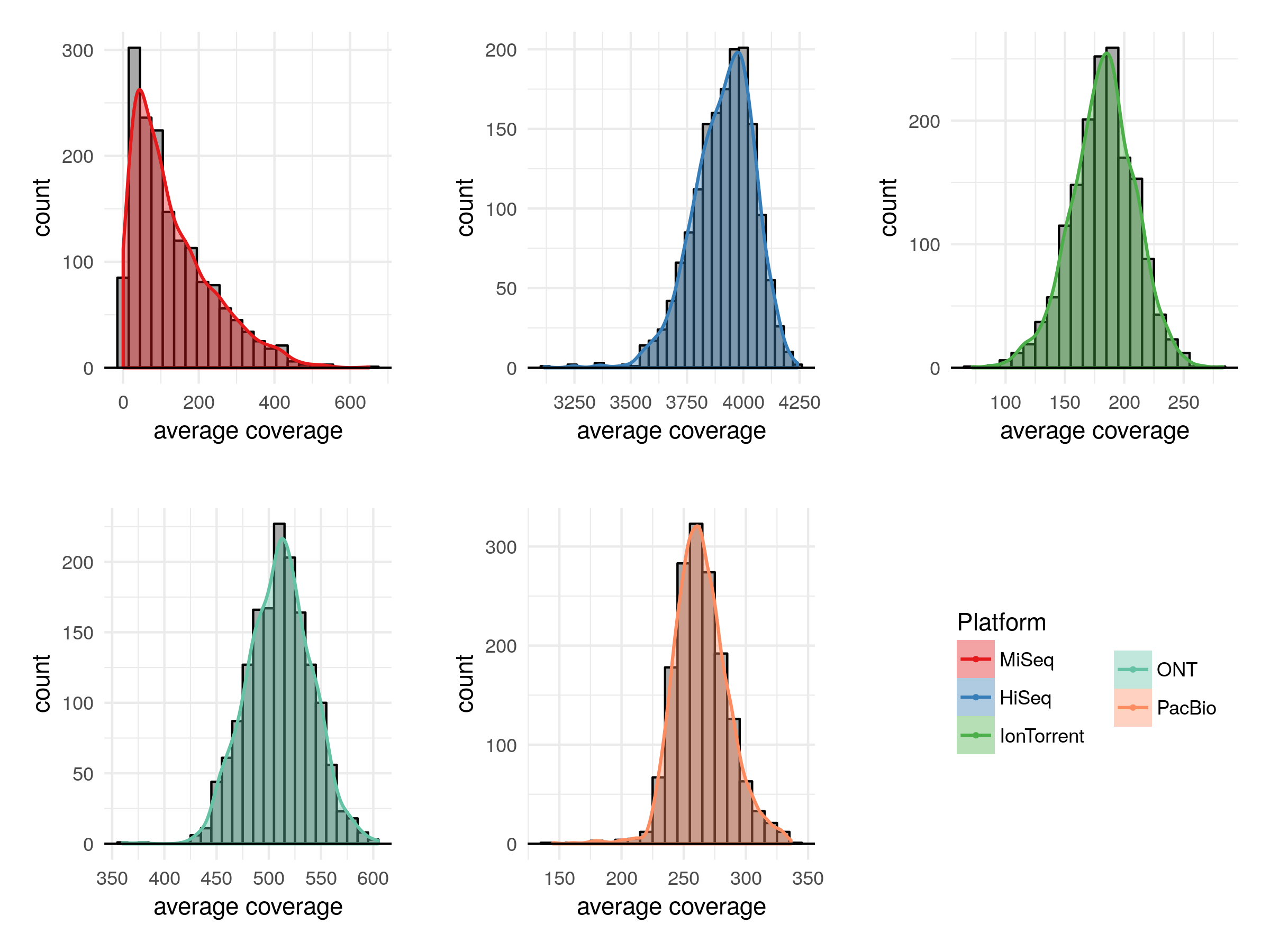

Supplement: Supplementary file 1 — Additional file 1: Supplementary Figure 1. Histograms of average coverage with density of gene regions for used sequencer platforms in FSC237 isolate. [file 12864_2021_8115_MOESM1_ESM.png]

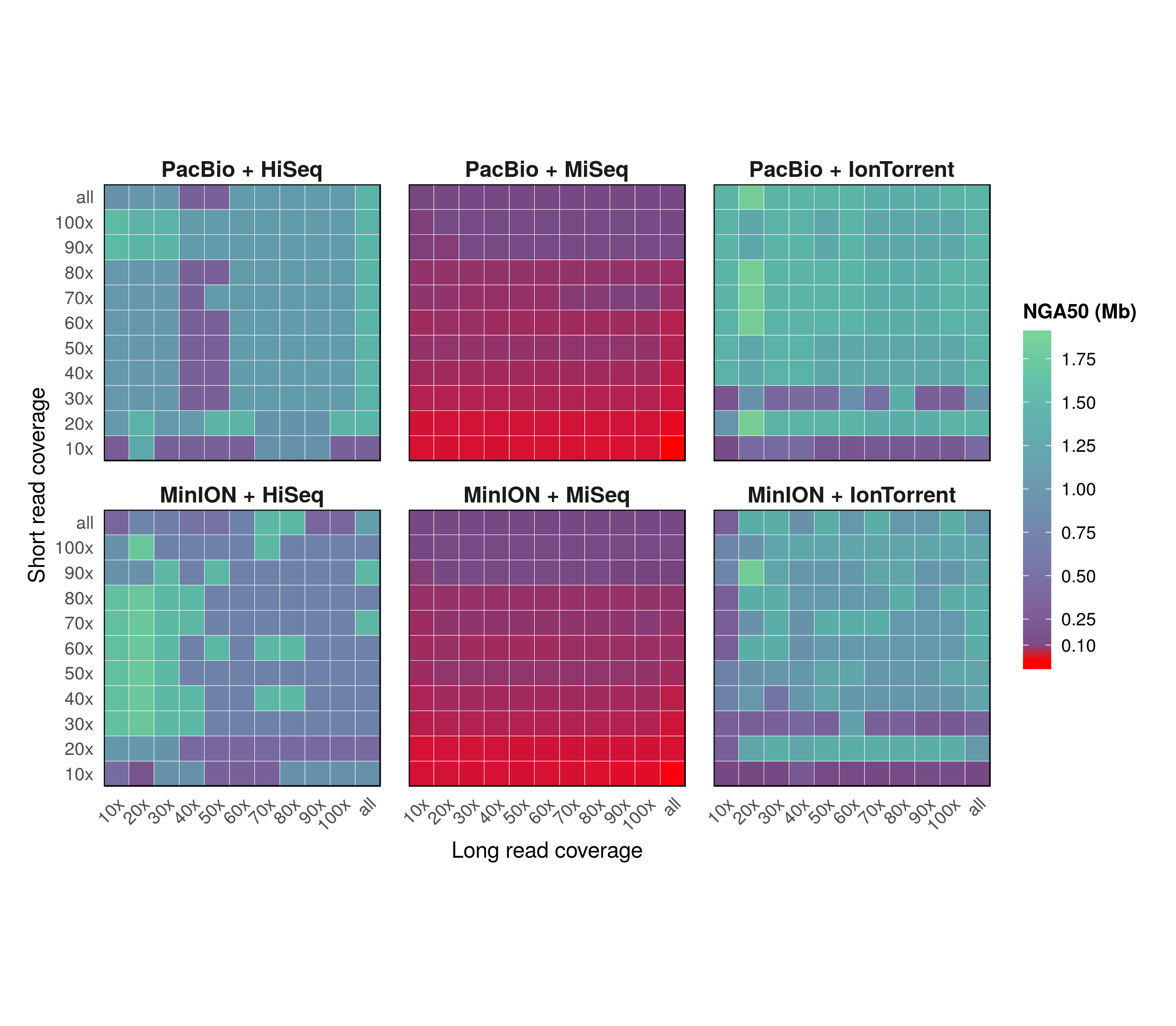

Supplement: Supplementary file 2 — Additional file 2: Supplementary Figure 2. Bivariate plot of log-transformed read length against base call quality with hexagonal bins and marginal histograms (NanoPlot) of raw reads a) PacBio and b) ONT. [file 12864_2021_8115_MOESM2_ESM.zip › Supp_Fig_12a.png]

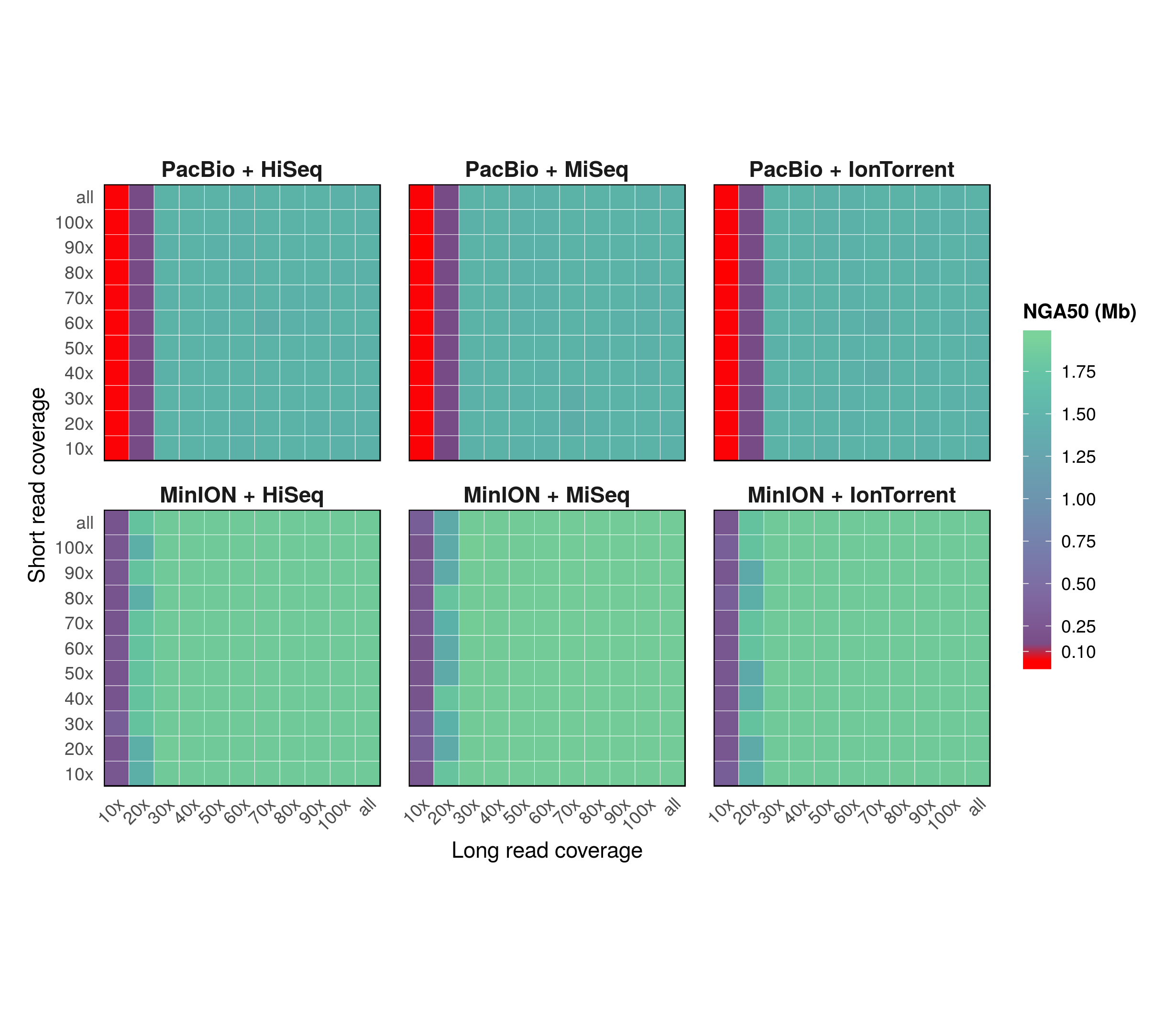

Supplement: Supplementary file 2 — Additional file 2: Supplementary Figure 2. Bivariate plot of log-transformed read length against base call quality with hexagonal bins and marginal histograms (NanoPlot) of raw reads a) PacBio and b) ONT. [file 12864_2021_8115_MOESM2_ESM.zip › Supp_Fig_12b.png]

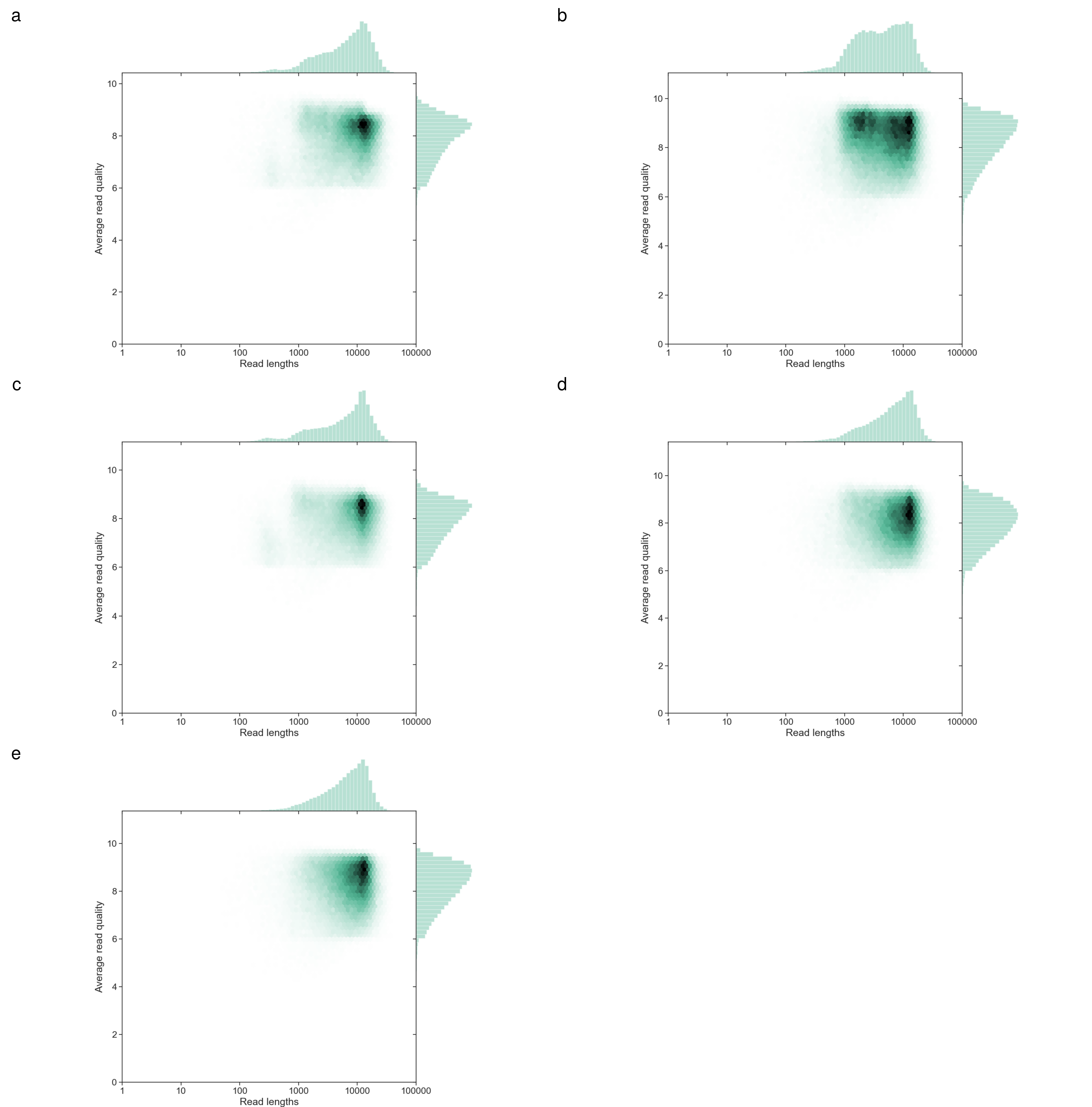

Supplement: Supplementary file 2 — Additional file 2: Supplementary Figure 2. Bivariate plot of log-transformed read length against base call quality with hexagonal bins and marginal histograms (NanoPlot) of raw reads a) PacBio and b) ONT. [file 12864_2021_8115_MOESM2_ESM.zip › Supp_Fig_2A.png]

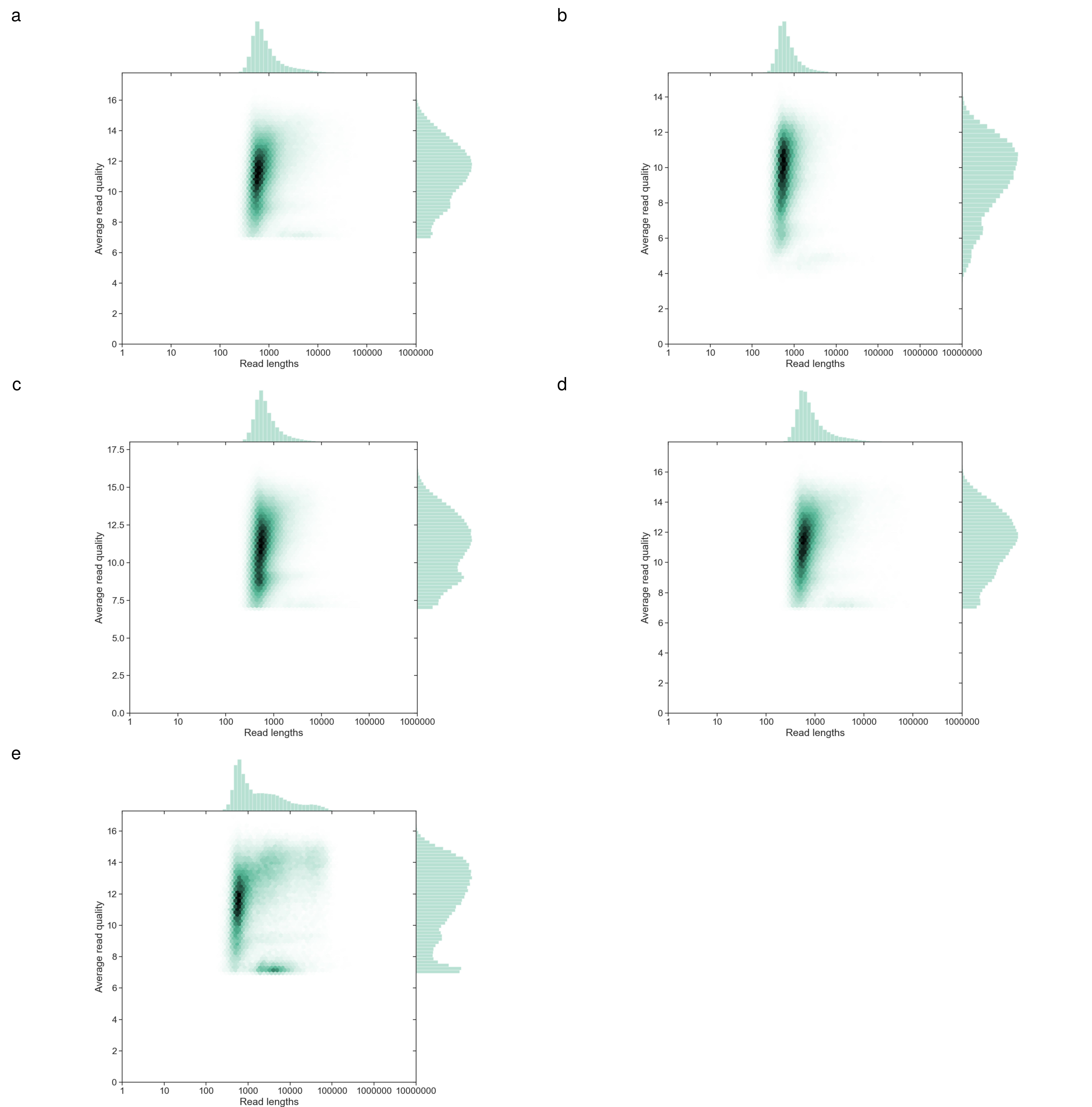

Supplement: Supplementary file 2 — Additional file 2: Supplementary Figure 2. Bivariate plot of log-transformed read length against base call quality with hexagonal bins and marginal histograms (NanoPlot) of raw reads a) PacBio and b) ONT. [file 12864_2021_8115_MOESM2_ESM.zip › Supp_Fig_2B.png]

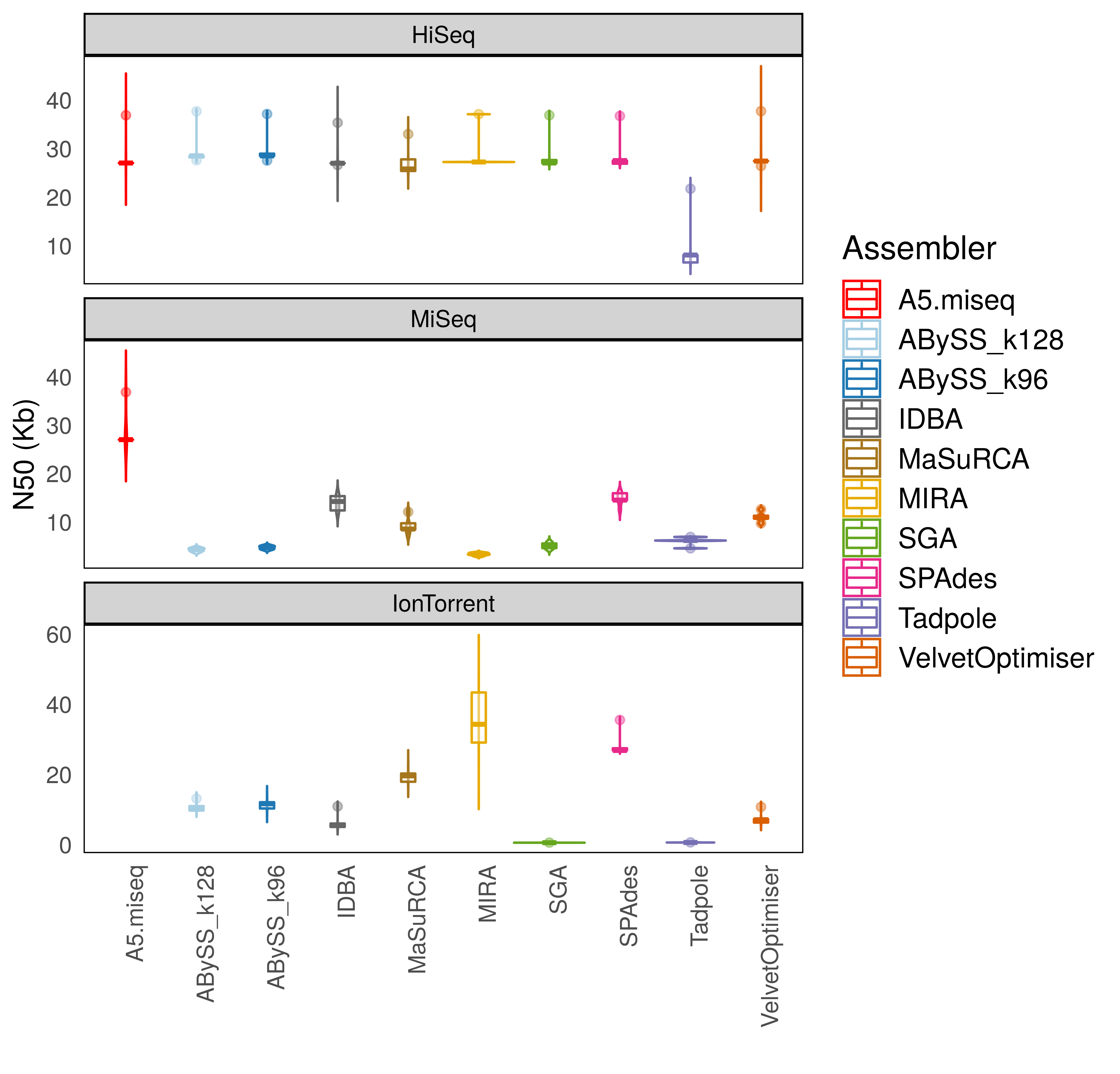

Supplement: Supplementary file 3 — Additional file 3: Supplementary Figure 3. Boxplots of N50 values for short-read assemblers. [file 12864_2021_8115_MOESM3_ESM.png]

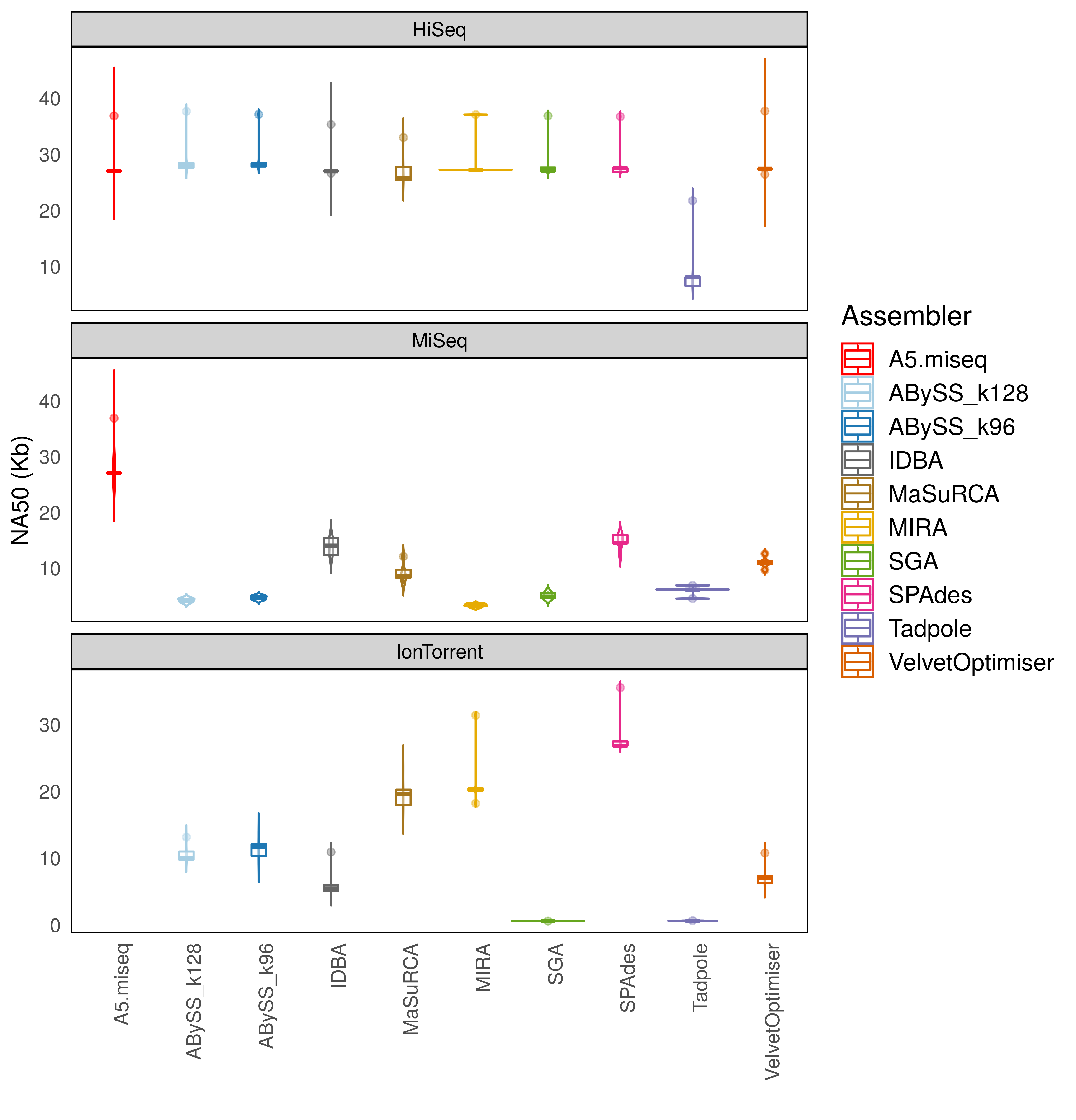

Supplement: Supplementary file 4 — Additional file 4: Supplementary Figure 4. Boxplots of NA50 values for short-read assemblers. [file 12864_2021_8115_MOESM4_ESM.png]

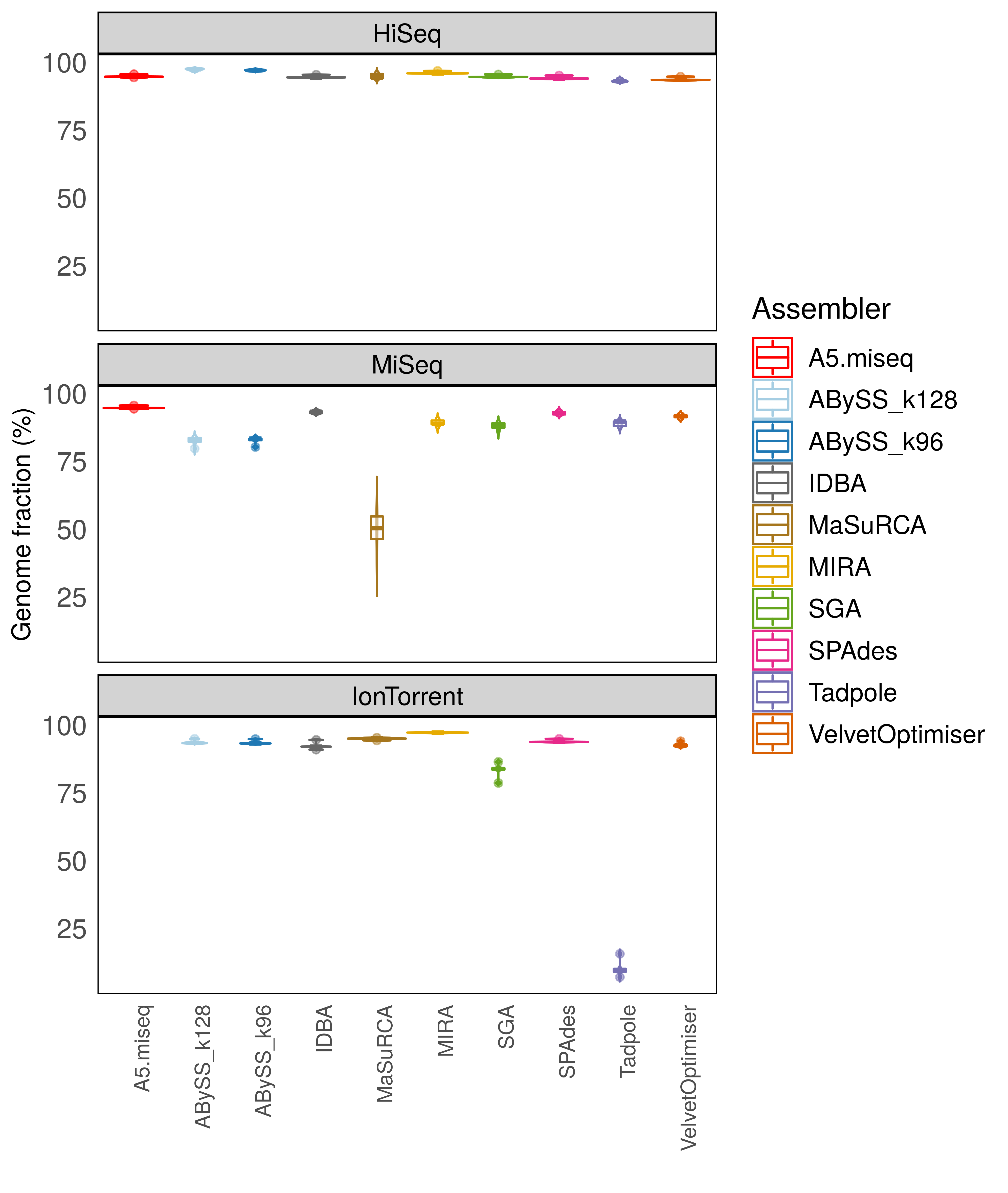

Supplement: Supplementary file 5 — Additional file 5: Supplementary Figure 5. Boxplots of genome fraction for all short-read assemblers. [file 12864_2021_8115_MOESM5_ESM.png]

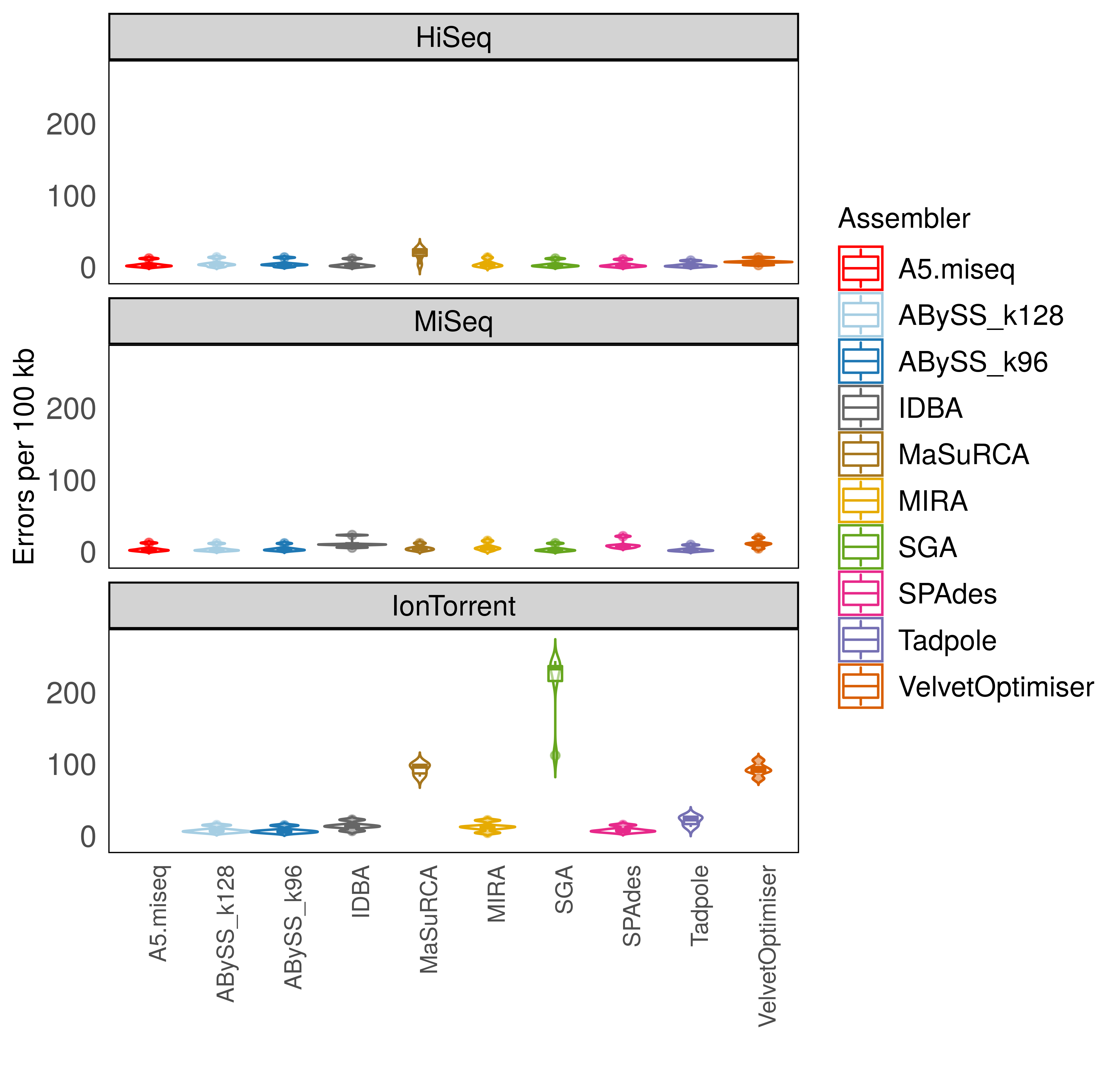

Supplement: Supplementary file 6 — Additional file 6: Supplementary Figure 6. Violin-Boxplots of assembly errors per 100 kb for all short-read assemblers. [file 12864_2021_8115_MOESM6_ESM.png]

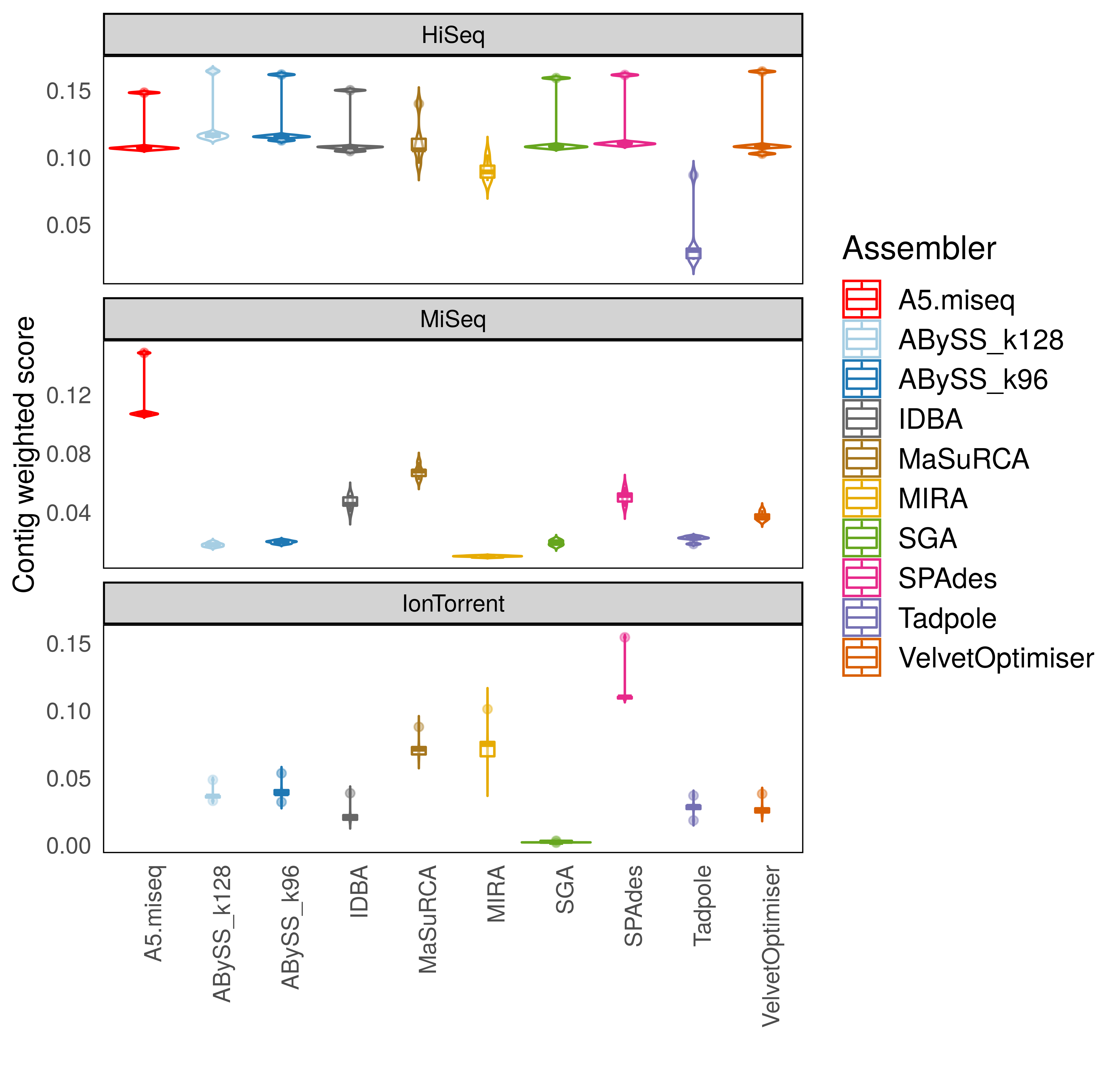

Supplement: Supplementary file 7 — Additional file 7: Supplementary Figure 7. Violin-Boxplots of contig weighted score for all short-read assemblers. [file 12864_2021_8115_MOESM7_ESM.png]

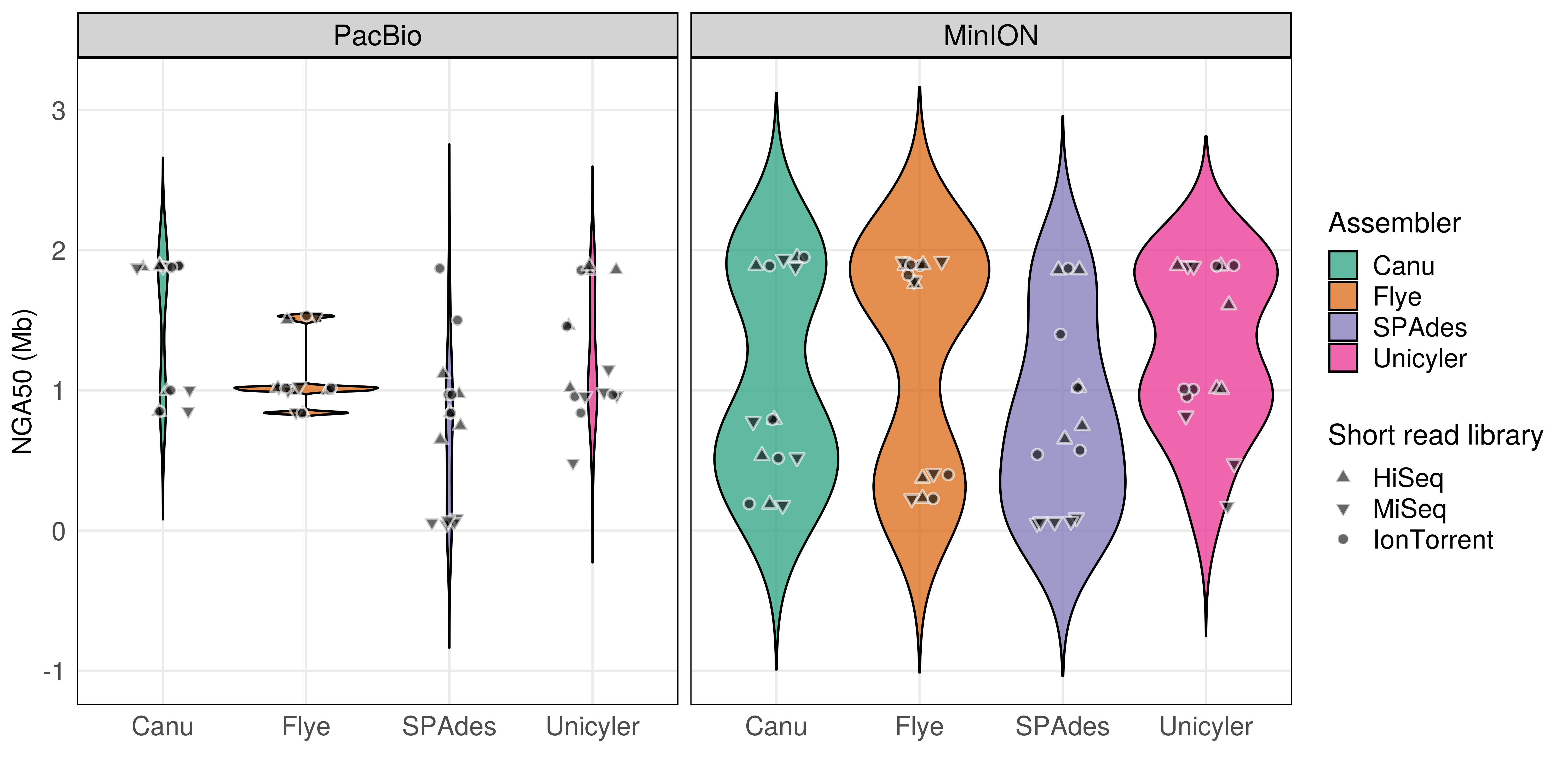

Supplement: Supplementary file 8 — Additional file 8: Supplementary Figure 8. Violin-Boxplots of NGA50 values for hybrid assemblies of all isolates. [file 12864_2021_8115_MOESM8_ESM.png]

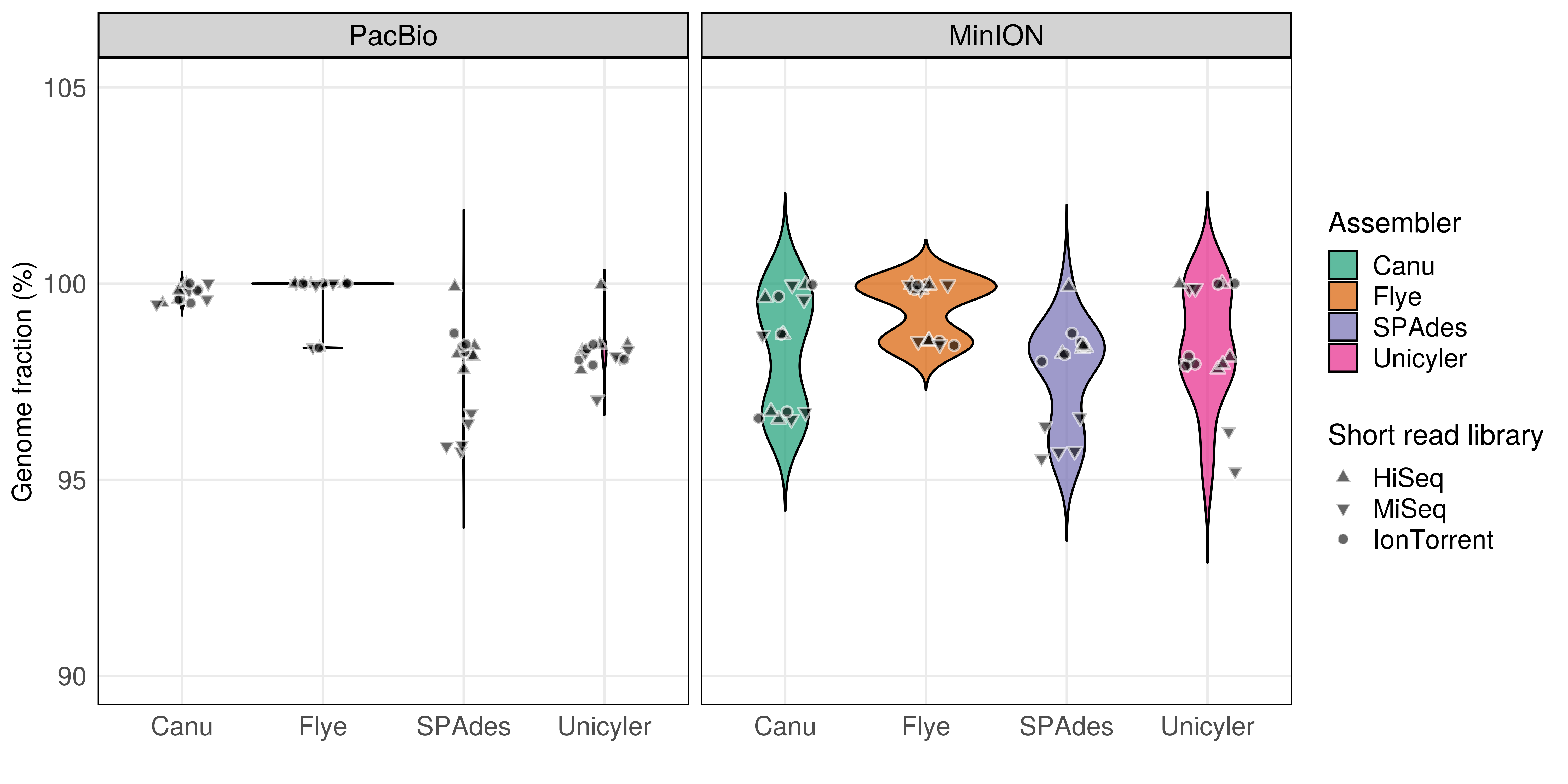

Supplement: Supplementary file 9 — Additional file 9: Supplementary Figure 9. Violin-Boxplots of genome fraction with respect to reference genomes for hybrid assembly of all isolates. [file 12864_2021_8115_MOESM9_ESM.png]

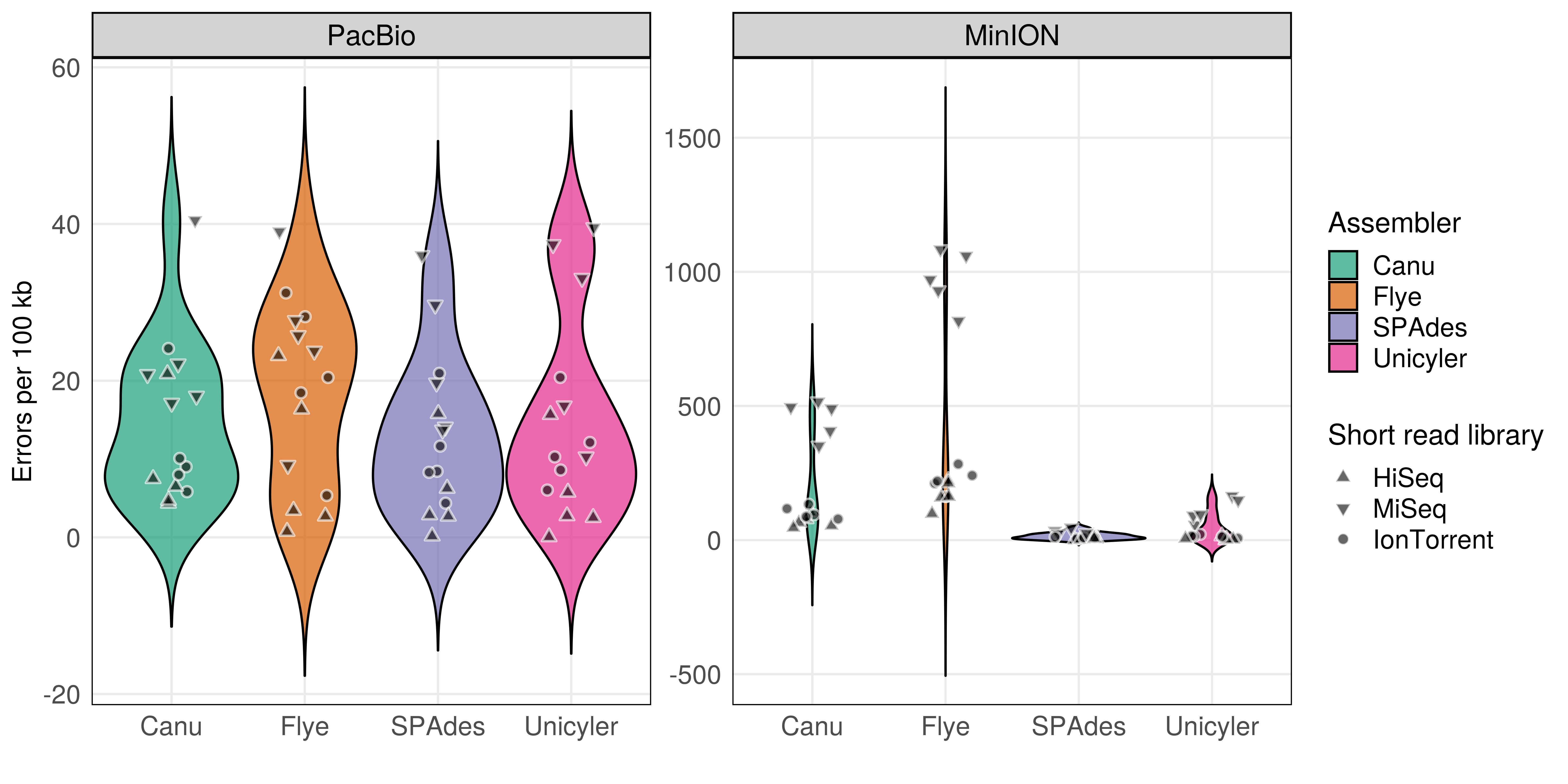

Supplement: Supplementary file 10 — Additional file 10: Supplementary Fig. 10. Violin-Boxplots of assembly errors per 100 kb for hybrid assembly of all isolates. [file 12864_2021_8115_MOESM10_ESM.png]

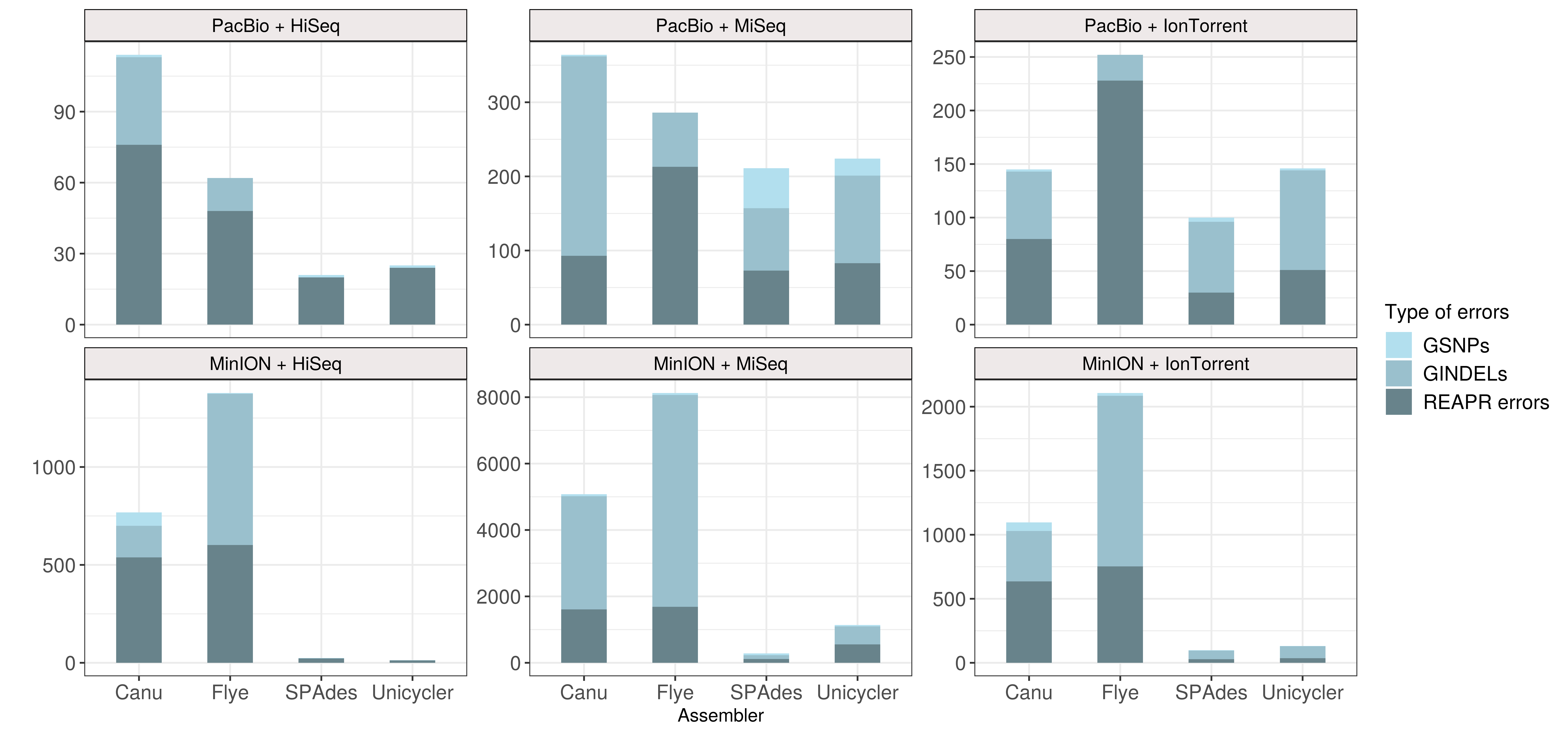

Supplement: Supplementary file 11 — Additional file 11: Supplementary Fig. 11. Break down of assembly errors in assembly of FSC237 in comparison to SCHU S4 reference (GSNPs, GIndels, REAPR errors ). The vertical axis indicates the total number of error in whole genome. [file 12864_2021_8115_MOESM11_ESM.png]

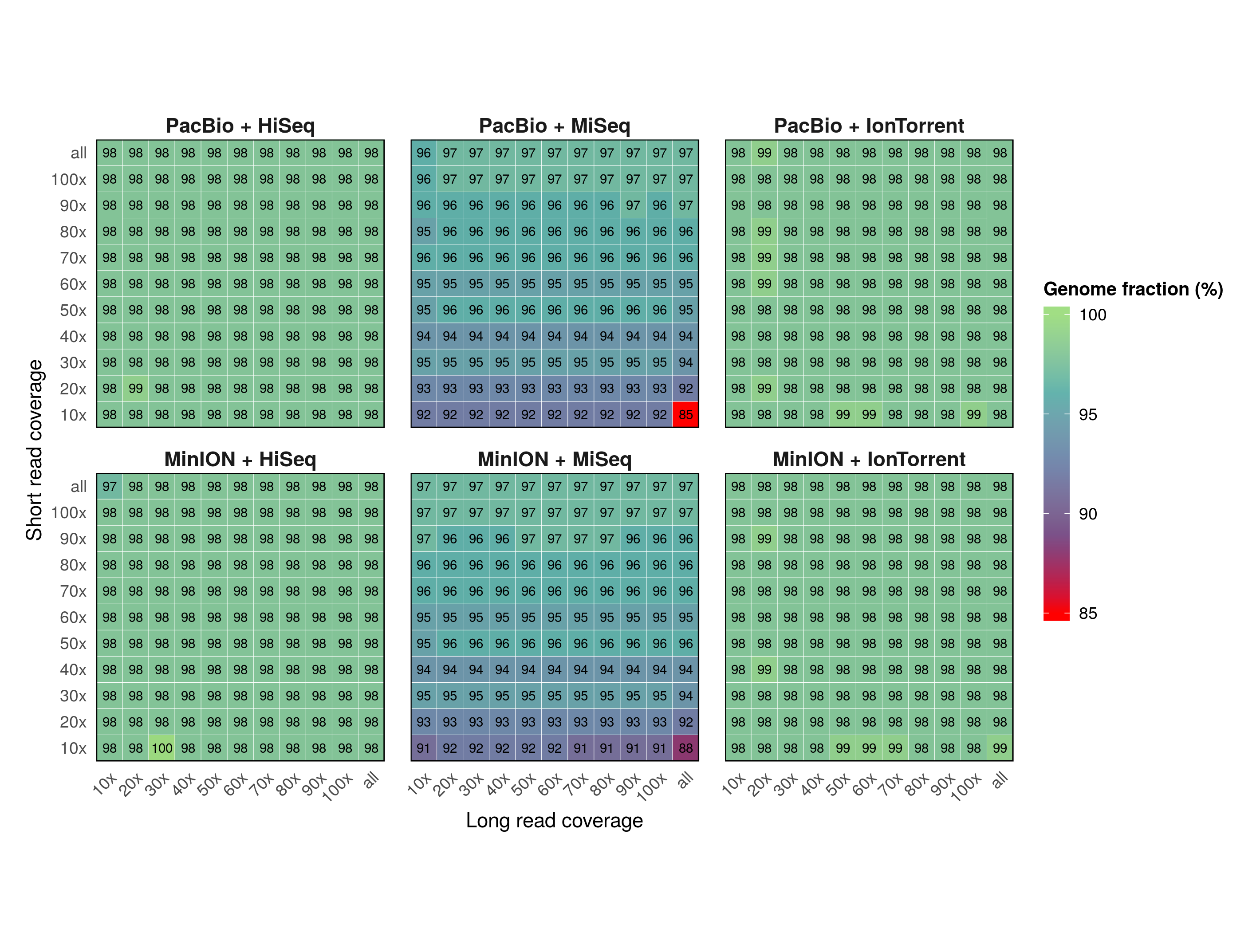

Supplement: Supplementary file 13 — Additional file 13: Supplementary Fig. 13. Effects of coverage on NGA50 values and errrors in Flye/P ilon assemblies. [file 12864_2021_8115_MOESM13_ESM.zip › Supp_Fig_13a.png]

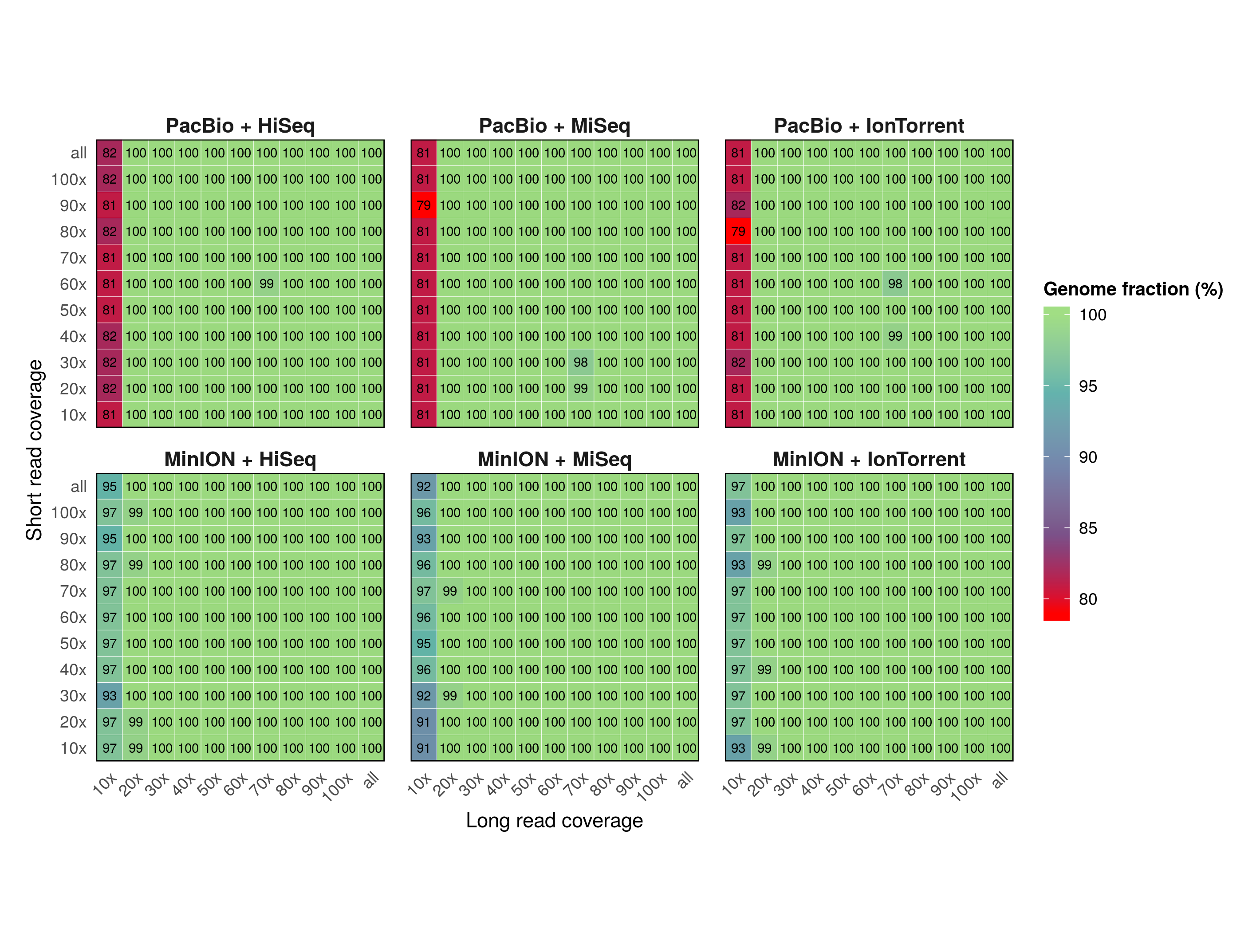

Supplement: Supplementary file 13 — Additional file 13: Supplementary Fig. 13. Effects of coverage on NGA50 values and errrors in Flye/P ilon assemblies. [file 12864_2021_8115_MOESM13_ESM.zip › Supp_Fig_13b.png]

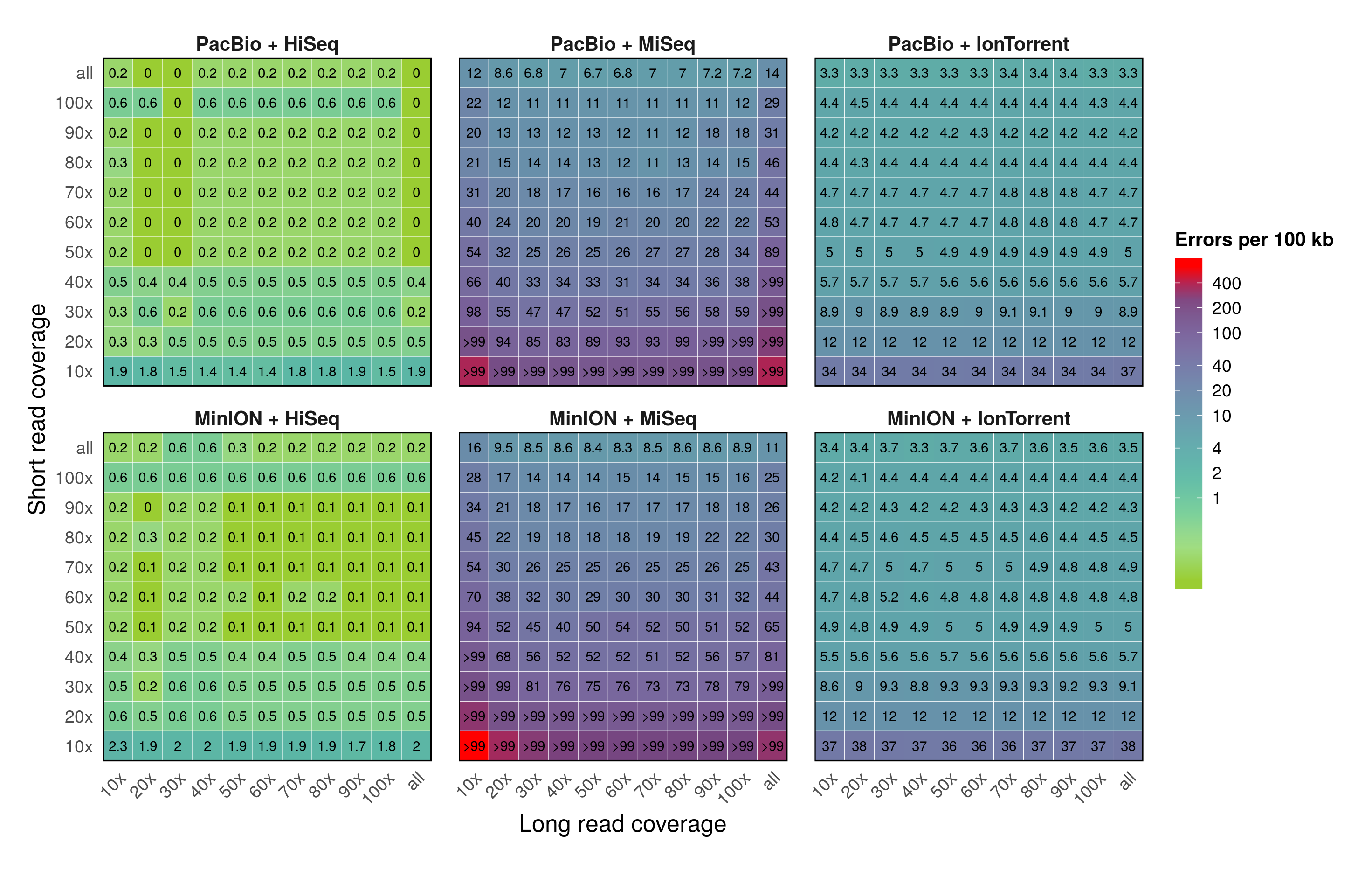

Supplement: Supplementary file 14 — Additional file 14: Supplementary Fig. 14. Effects of preprocessing on NGA50 and genome fraction in hybrid assemblies. [file 12864_2021_8115_MOESM14_ESM.zip › Supp_Fig_14a.png]

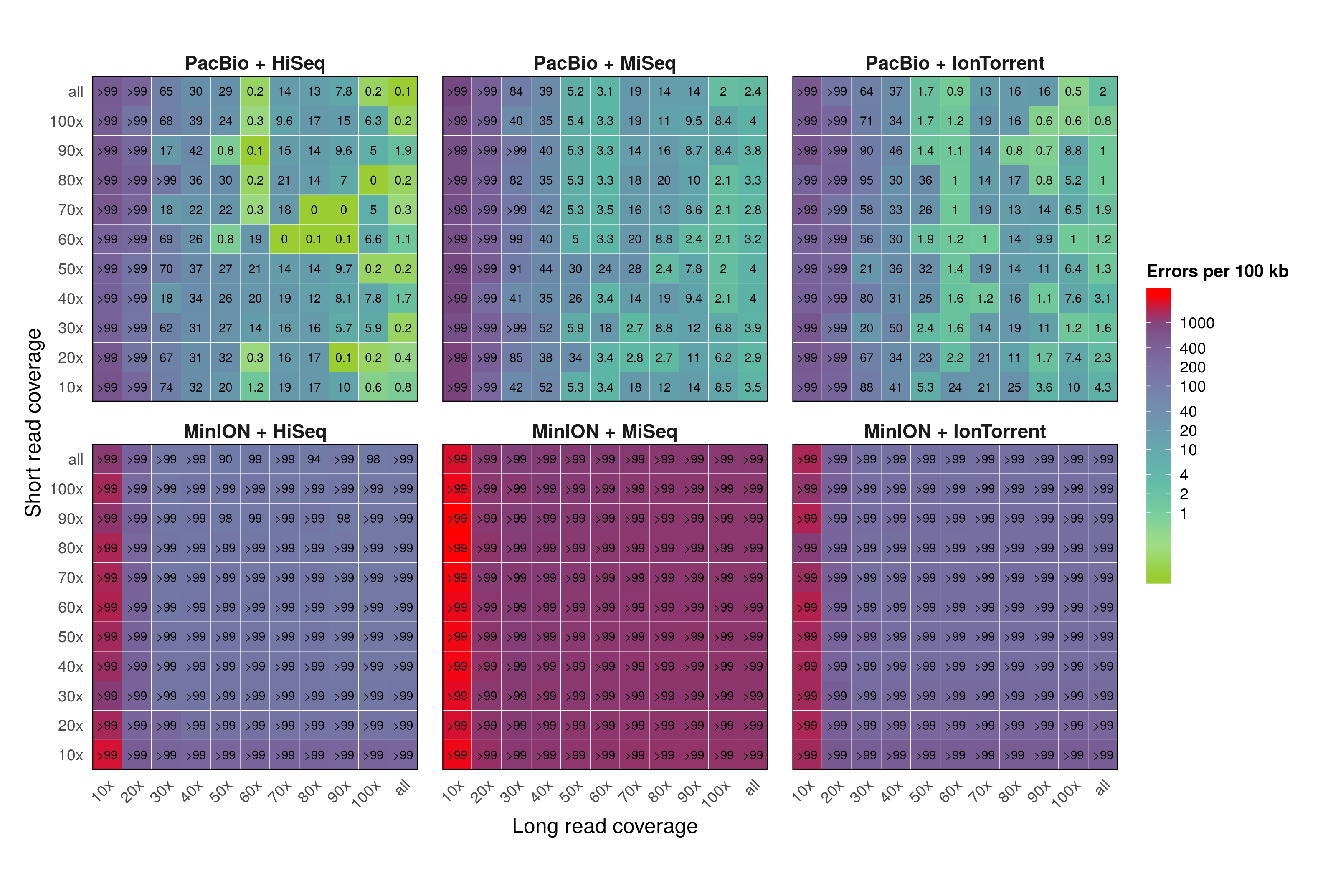

Supplement: Supplementary file 14 — Additional file 14: Supplementary Fig. 14. Effects of preprocessing on NGA50 and genome fraction in hybrid assemblies. [file 12864_2021_8115_MOESM14_ESM.zip › Supp_Fig_14b.png]

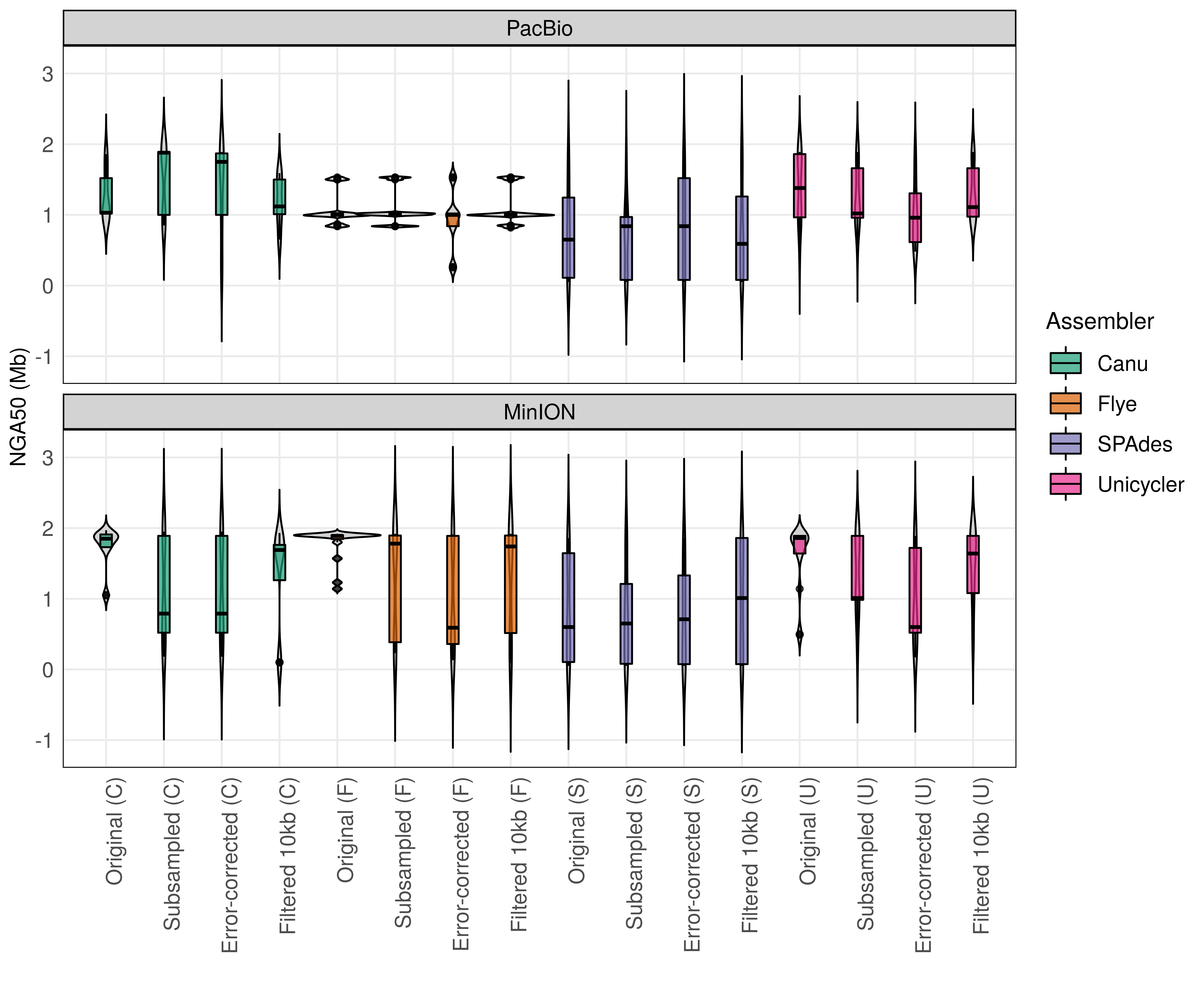

Supplement: Supplementary file 15 — Additional file 15: Supplementary Fig. 15. Effects of preprocessing on errors in hybrid assembl ies. [file 12864_2021_8115_MOESM15_ESM.zip › Supp_Fig_15a.png]

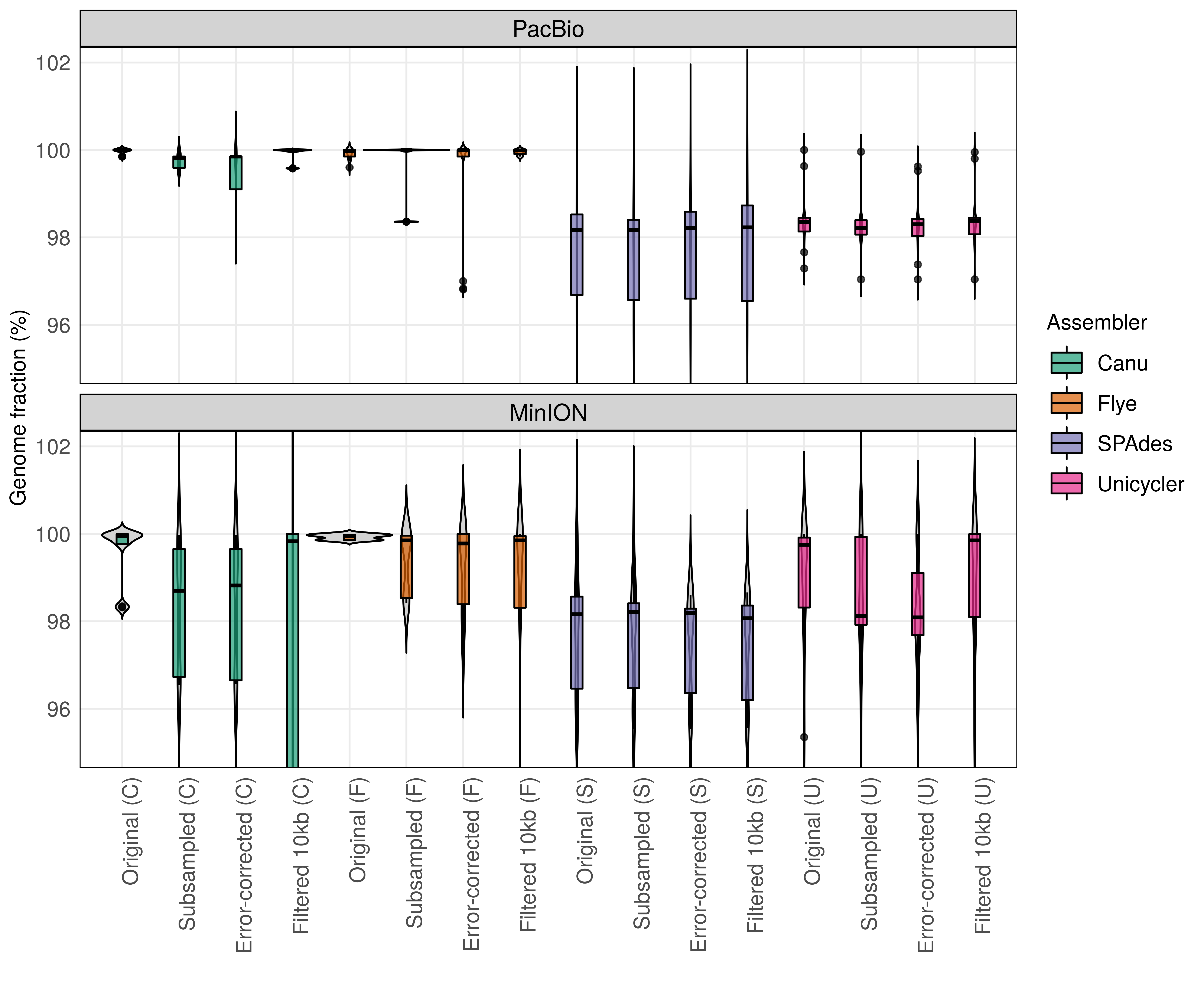

Supplement: Supplementary file 15 — Additional file 15: Supplementary Fig. 15. Effects of preprocessing on errors in hybrid assembl ies. [file 12864_2021_8115_MOESM15_ESM.zip › Supp_Fig_15b.png]

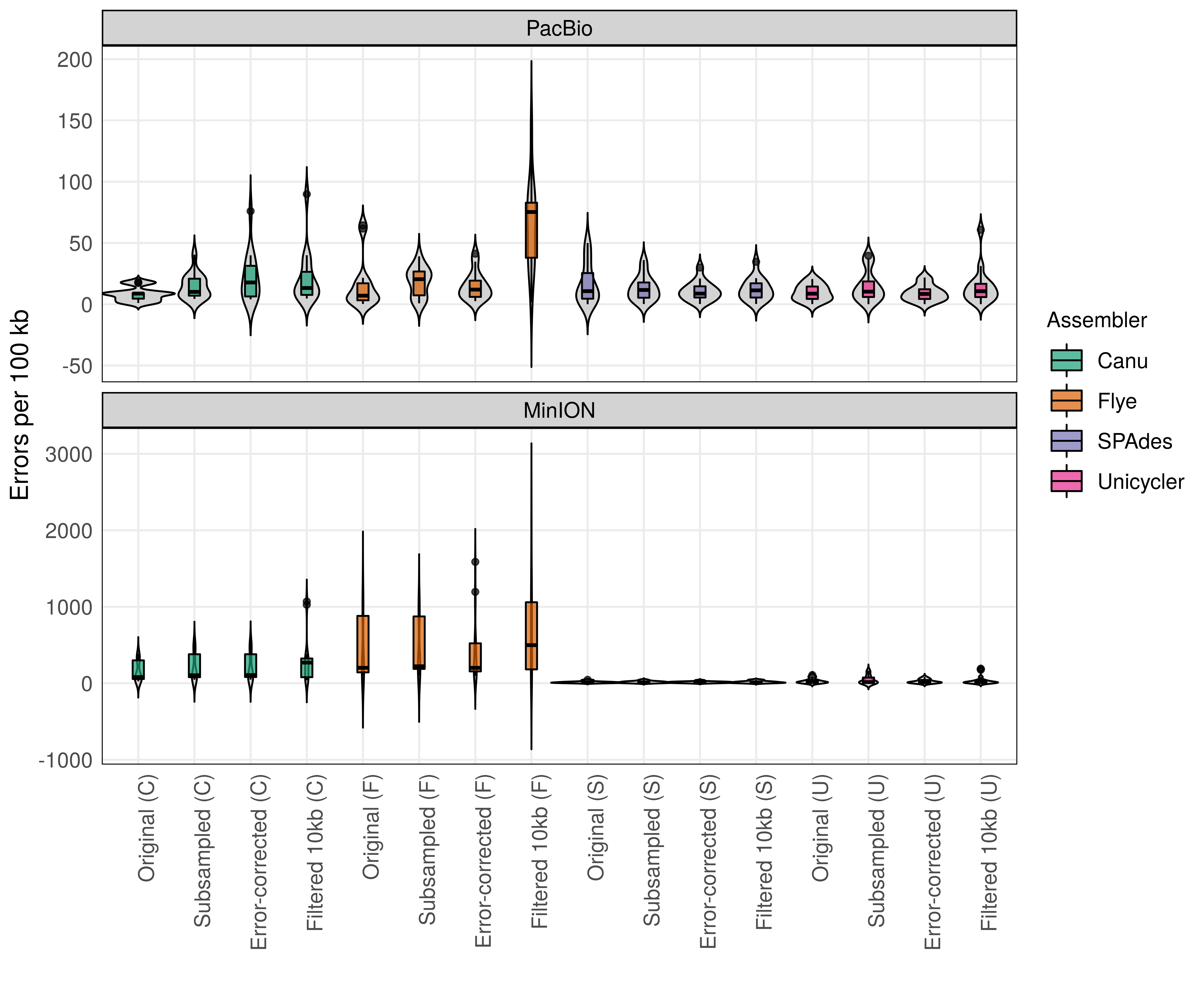

Supplement: Supplementary file 15 — Additional file 15: Supplementary Fig. 15. Effects of preprocessing on errors in hybrid assembl ies. [file 12864_2021_8115_MOESM15_ESM.zip › Supp_Fig_15c.png]

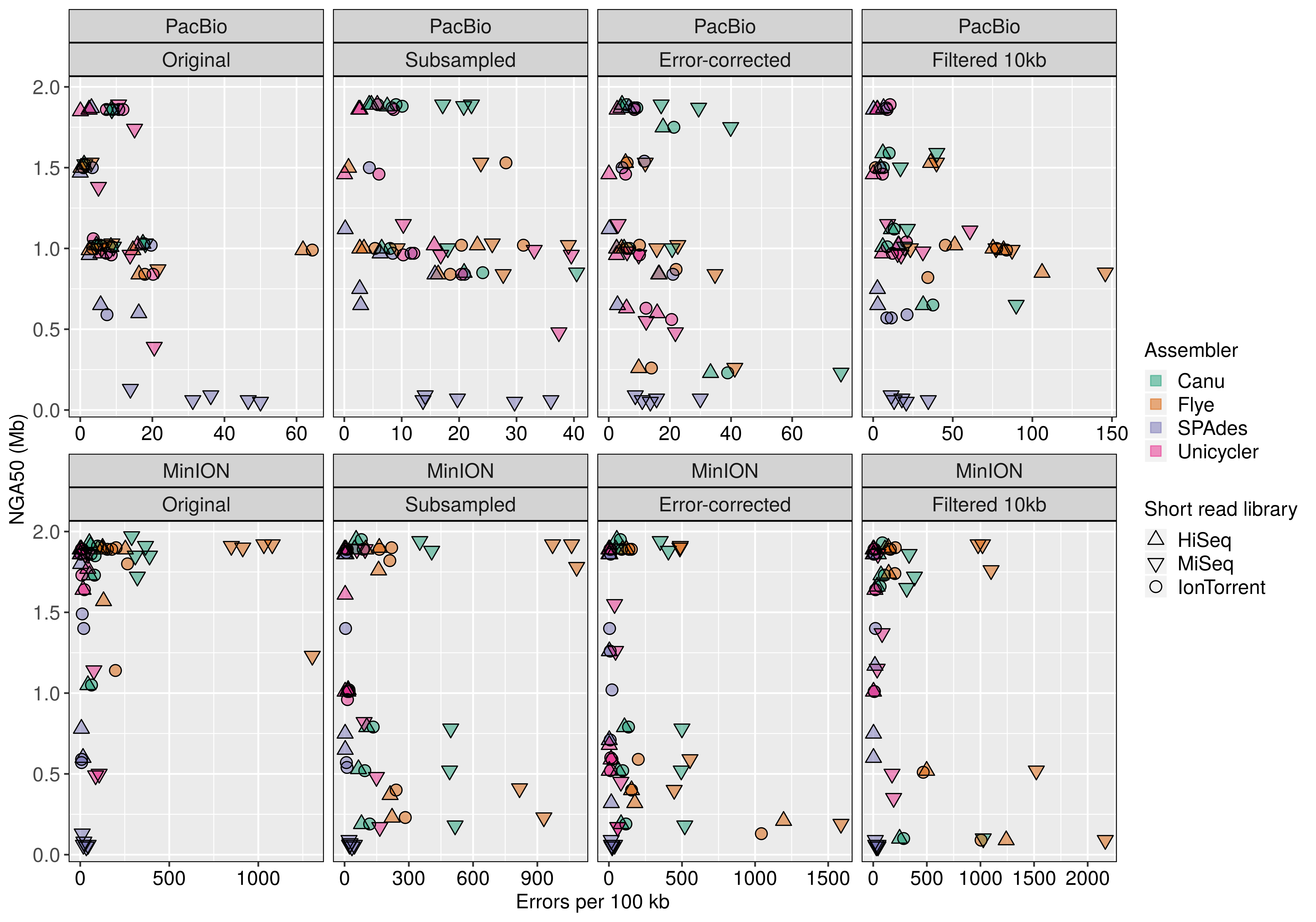

Supplement: Supplementary file 16 — Additional file 16: Supplementary Fig. 16. NGA50 values versus assembly errors for vari ous prepr ocessing methods in hybrid assemblies. [file 12864_2021_8115_MOESM16_ESM.png]
