# Supplementary material for: Testing assembly strategies of Francisella tularensis genomes to infer an evolutionary conservation analysis of genomic structures
Source: BMC Genomics. 2021 Nov 14;22:822. doi: 10.1186/s12864-021-08115-x (PMC8590783; doi:10.1186/s12864-021-08115-x)
Supplement: Supplementary file 17 — Additional file 17: Supplementary Fig. 17. Sequence alignments (green) with Francisella pathogenicity islands (blue) revealed them as a major cause for misassembly. To prevent Blast from generating artifacts, the minimum alignment length parameter were set to > 40 kb in Bandage or FPI sequences were masked prior to Blast. [file 12864_2021_8115_MOESM17_ESM.pdf]

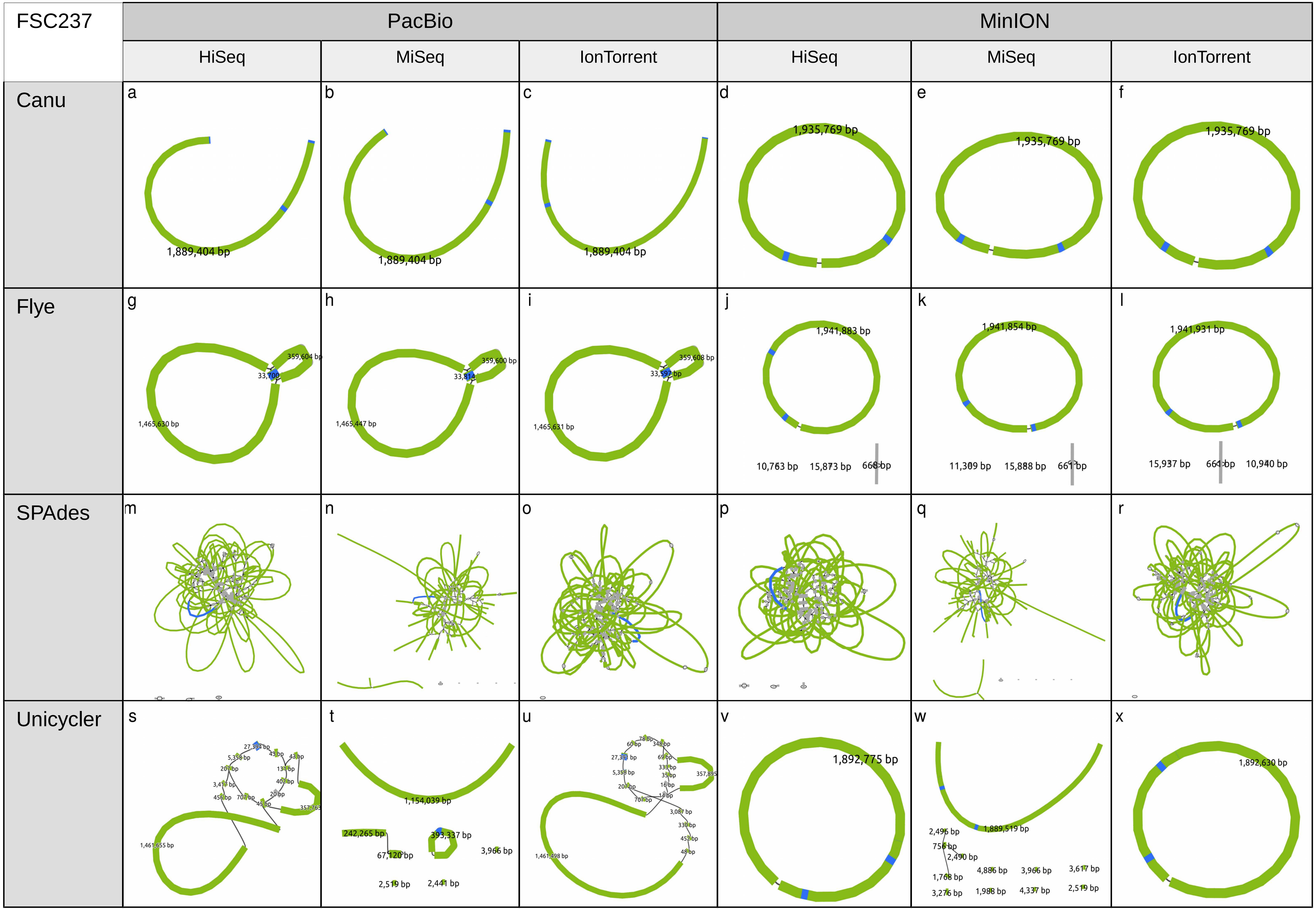

FSC237
PacBio
MinION
HiSeq
MiSeq
IonTorrent
HiSeq
MiSeq
IonTorrent
Canu
Flye
SPAdes
Unicycler
a
1,889,404 bp
b
1,889,404 bp
c
1,889,404 bp
d
1,935,769 bp
e
1,935,769 bp
f
1,935,769 bp
g
359,604 bp
33,700
1,465,630 bp
h
359,600 bp
33,814
1,465,447 bp
i
359,608 bp
1,465,631 bp
j
1,941,883 bp
10,763 bp
15,873 bp
668 bp
k
1,941,854 bp
11,309 bp
15,888 bp
661 bp
l
1,941,931 bp
15,937 bp
661 bp
10,940 bp
m
n
o
p
q
r
s
1,461,655 bp
t
1,154,039 bp
242,265 bp
393,337 bp
67,120 bp
3,966 bp
2,519 bp
2,441 bp
u
1,461,498 bp
v
1,892,775 bp
w
1,889,519 bp
2,496 bp
756 bp
2,490 bp
4,886 bp
3,966 bp
3,617 bp
1,768 bp
3,276 bp
1,988 bp
4,337 bp
2,519 bp
x
1,892,630 bp

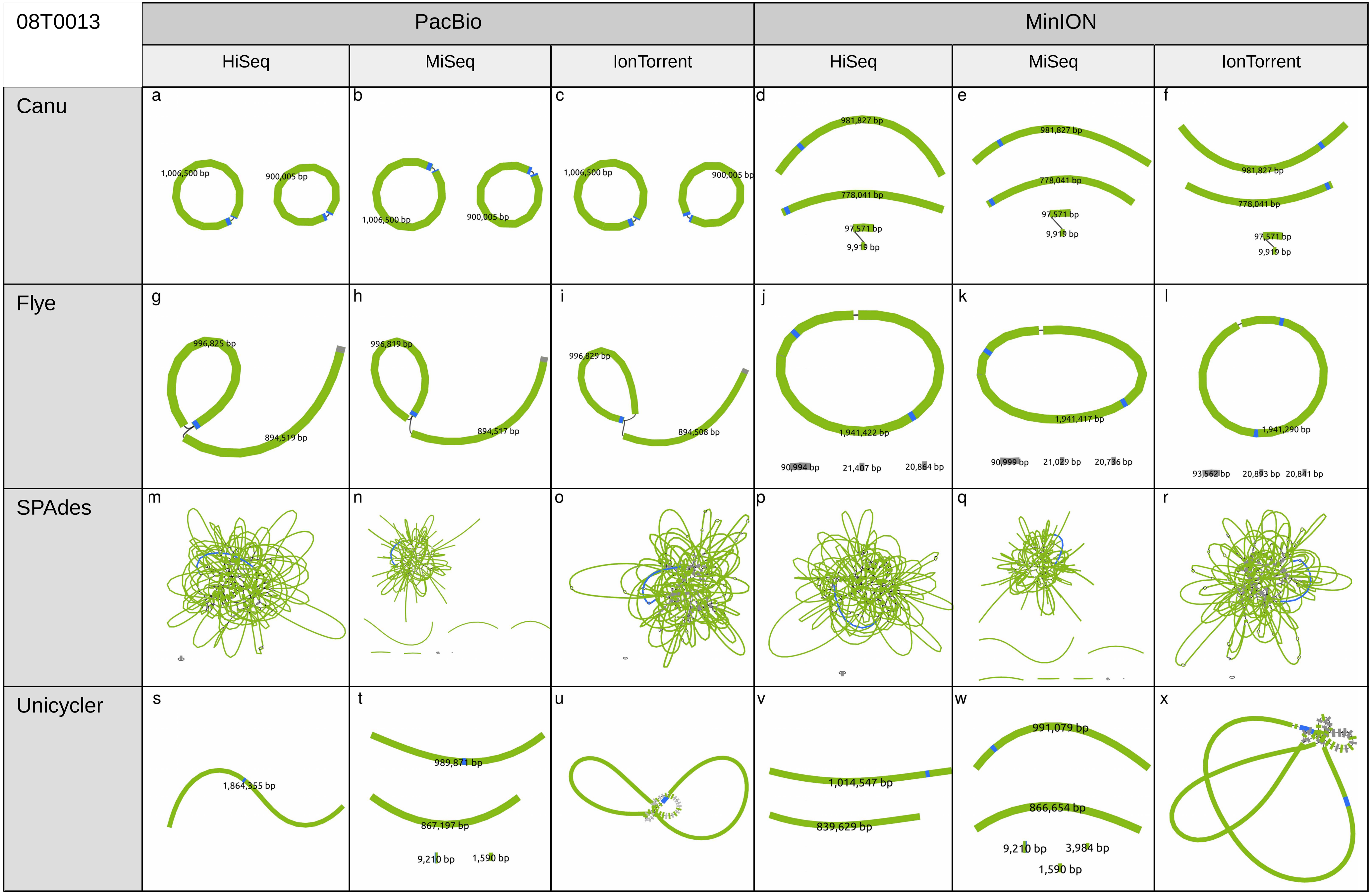

08T0013
PacBio
MinION
HiSeq
MiSeq
IonTorrent
HiSeq
MiSeq
IonTorrent
Canu
a
1,006,500 bp
900,005 bp
b
1,006,500 bp
900,005 bp
c
1,006,500 bp
900,005 bp
d
981,827 bp
778,041 bp
97,571 bp
9,919 bp
e
981,827 bp
778,041 bp
97,571 bp
9,919 bp
f
981,827 bp
778,041 bp
97,571 bp
9,919 bp
Flye
g
996,825 bp
894,519 bp
h
996,819 bp
894,517 bp
i
996,829 bp
894,508 bp
j
1,941,422 bp
90,994 bp
21,407 bp
20,864 bp
k
1,941,417 bp
90,999 bp
21,029 bp
20,736 bp
l
1,941,290 bp
93,562 bp
20,893 bp
20,841 bp
SPAdes
m
n
o
p
q
r
Unicycler
s
1,864,355 bp
t
989,871 bp
867,197 bp
9,210 bp
1,590 bp
u
v
1,014,547 bp
839,629 bp
w
991,079 bp
866,654 bp
9,210 bp
3,984 bp
1,590 bp
x

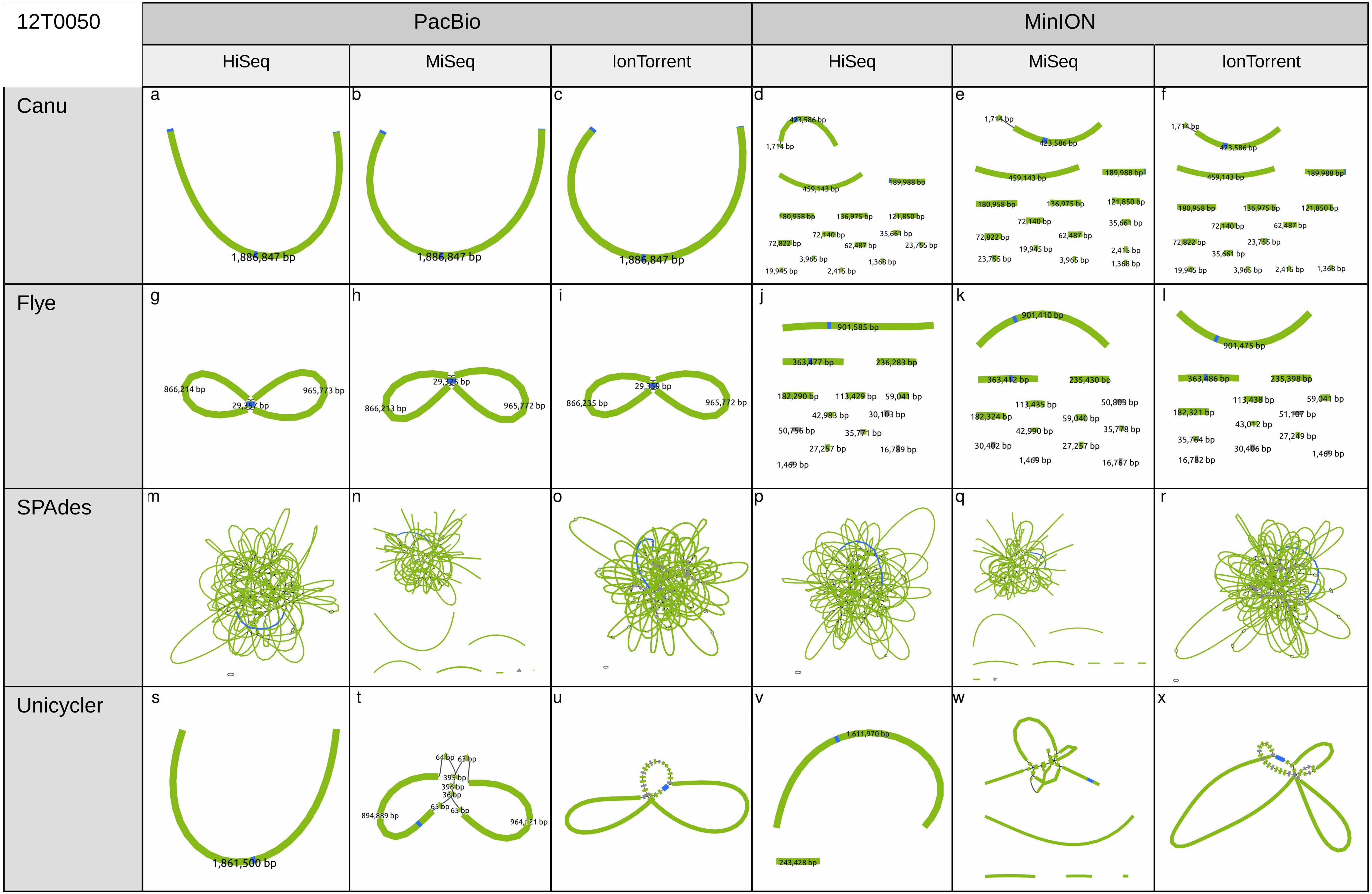
12T0050
PacBio
MinION
HiSeq
MiSeq
IonTorrent
HiSeq
MiSeq
IonTorrent
Canu
Flye
SPAdes
Unicycler
a
1,886,847 bp
b
1,886,847 bp
c
1,886,847 bp
d
423,586 bp
1,714 bp
459,143 bp
189,988 bp
180,958 bp
136,975 bp
121,850 bp
72,140 bp
35,661 bp
72,822 bp
62,487 bp
23,755 bp
3,965 bp
1,368 bp
19,945 bp
2,415 bp
e
1,714 bp
423,586 bp
459,143 bp
189,988 bp
180,958 bp
136,975 bp
121,850 bp
72,140 bp
35,661 bp
72,822 bp
62,487 bp
19,945 bp
2,415 bp
23,755 bp
3,965 bp
1,368 bp
f
1,714 bp
423,586 bp
459,143 bp
189,988 bp
180,958 bp
136,975 bp
121,850 bp
72,140 bp
62,487 bp
72,822 bp
23,755 bp
35,661 bp
19,945 bp
3,965 bp
2,415 bp
1,368 bp
g
866,214 bp
29,357 bp
965,773 bp
h
29,325 bp
866,213 bp
965,772 bp
i
29,359 bp
866,235 bp
965,772 bp
j
901,585 bp
363,477 bp
236,283 bp
182,290 bp
113,429 bp
59,041 bp
42,983 bp
30,103 bp
50,756 bp
35,771 bp
27,257 bp
16,789 bp
1,469 bp
k
901,410 bp
363,412 bp
235,430 bp
113,435 bp
50,803 bp
182,324 bp
59,040 bp
42,990 bp
35,778 bp
30,402 bp
27,257 bp
1,469 bp
16,767 bp
l
901,475 bp
363,486 bp
235,398 bp
113,438 bp
59,041 bp
182,321 bp
51,107 bp
43,012 bp
27,249 bp
35,764 bp
30,406 bp
1,469 bp
16,782 bp
m
n
o
p
q
r
s
1,861,500 bp
t
64 bp
63 bp
395 bp
396 bp
36 bp
65 bp
65 bp
894,889 bp
964,121 bp
u
v
1,611,970 bp
243,428 bp
w
x

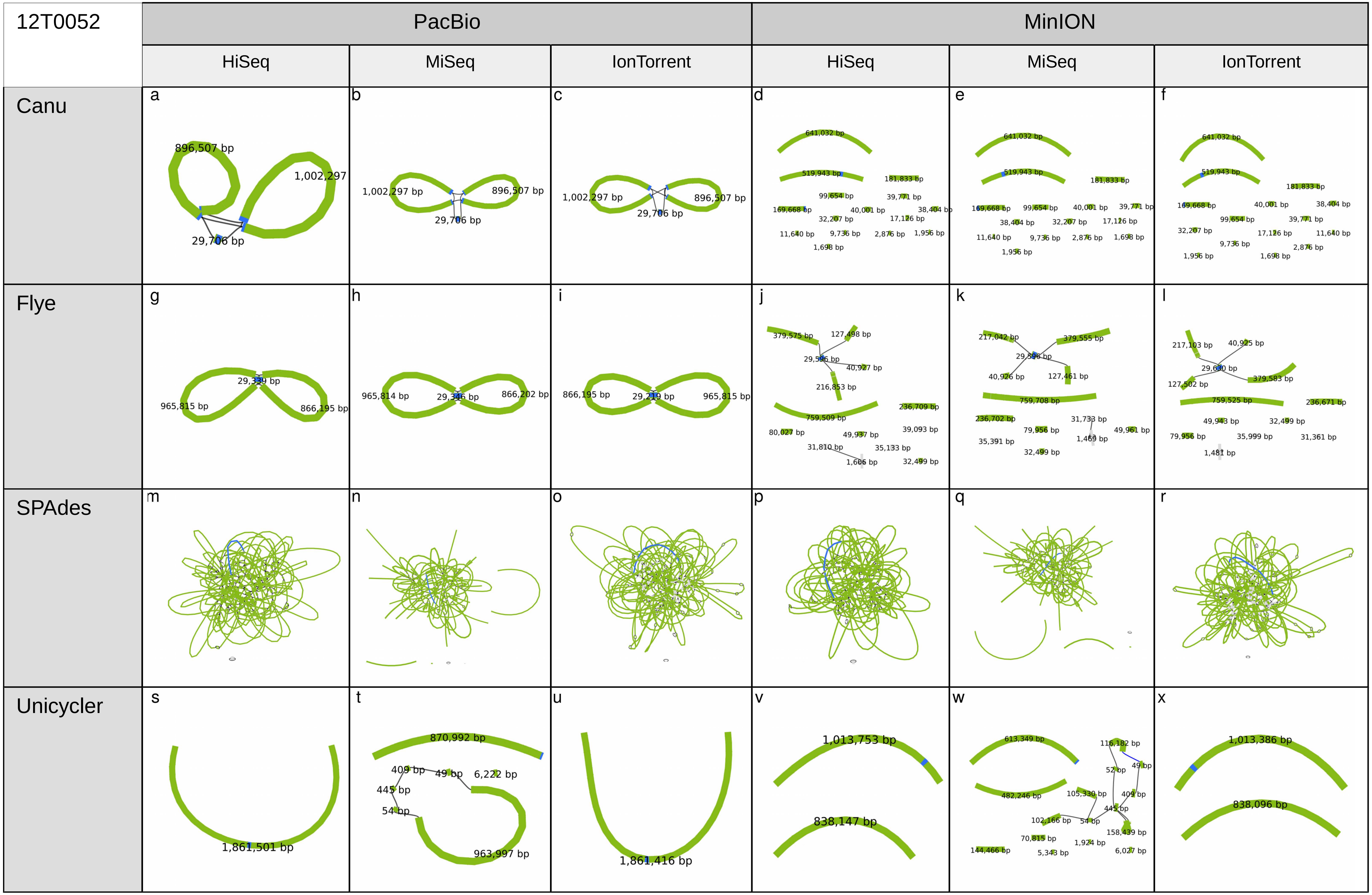

12T0052
PacBio
MinION
HiSeq
MiSeq
IonTorrent
HiSeq
MiSeq
IonTorrent
Canu
a
896,507 bp
1,002,297
29,706 bp
b
1,002,297 bp
896,507 bp
29,706 bp
c
1,002,297 bp
896,507 bp
29,706 bp
d
641,032 bp
519,943 bp
181,833 bp
99,654 bp
39,771 bp
169,668 bp
40,001 bp
38,404 bp
32,207 bp
17,126 bp
11,640 bp
9,736 bp
2,876 bp
1,956 bp
1,698 bp
e
641,032 bp
519,943 bp
181,833 bp
169,668 bp
99,654 bp
40,001 bp
39,771 bp
38,404 bp
32,207 bp
17,126 bp
11,640 bp
9,736 bp
2,876 bp
1,698 bp
1,956 bp
f
641,032 bp
519,943 bp
181,833 bp
169,668 bp
40,001 bp
38,404 bp
99,654 bp
39,771 bp
32,207 bp
17,126 bp
11,640 bp
9,736 bp
2,876 bp
1,956 bp
1,698 bp
Flye
g
29,339 bp
965,815 bp
866,195 bp
h
965,814 bp
29,346 bp
866,202 bp
i
866,195 bp
29,219 bp
965,815 bp
j
379,575 bp
127,498 bp
29,596 bp
40,927 bp
216,853 bp
759,509 bp
236,709 bp
80,027 bp
49,937 bp
39,093 bp
31,810 bp
35,133 bp
1,606 bp
32,499 bp
k
217,042 bp
379,555 bp
29,596 bp
40,926 bp
127,461 bp
759,708 bp
236,702 bp
31,733 bp
79,956 bp
49,961 bp
35,391 bp
1,469 bp
32,499 bp
l
217,103 bp
40,925 bp
29,630 bp
127,502 bp
379,583 bp
759,525 bp
236,671 bp
49,943 bp
32,499 bp
79,956 bp
35,999 bp
31,361 bp
1,481 bp
SPAdes
m
n
o
p
q
r
Unicycler
s
1,861,501 bp
t
870,992 bp
409 bp
49 bp
6,222 bp
445 bp
54 bp
963,997 bp
u
1,861,416 bp
v
1,013,753 bp
838,147 bp
w
613,349 bp
116,182 bp
52 bp
49 bp
482,246 bp
105,330 bp
409 bp
445 bp
102,166 bp
54 bp
158,439 bp
70,815 bp
1,924 bp
144,466 bp
5,343 bp
6,027 bp
x
1,013,386 bp
838,096 bp

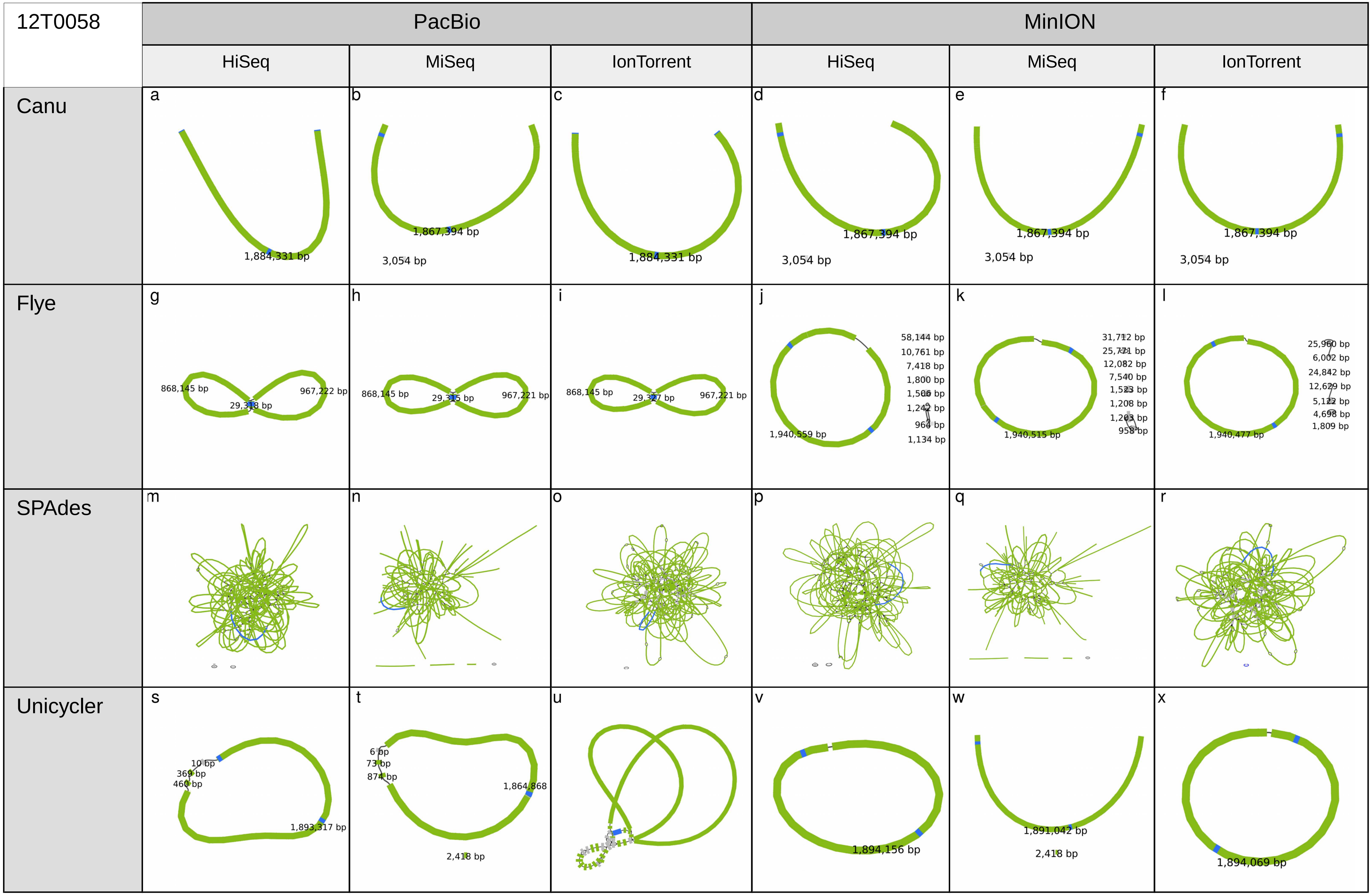

12T0058
PacBio
MinION
HiSeq
MiSeq
IonTorrent
HiSeq
MiSeq
IonTorrent
Canu
a
1,884,331 bp
b
1,867,394 bp
3,054 bp
c
1,884,331 bp
d
1,867,394 bp
3,054 bp
e
1,867,394 bp
3,054 bp
f
1,867,394 bp
3,054 bp
Flye
g
868,145 bp
29,318 bp
967,222 bp
h
868,145 bp
29,315 bp
967,221 bp
i
868,145 bp
29,327 bp
967,221 bp
j
58,144 bp
10,761 bp
7,418 bp
1,800 bp
1,506 bp
1,242 bp
964 bp
1,134 bp
1,940,559 bp
k
31,712 bp
25,771 bp
12,082 bp
7,540 bp
1,523 bp
1,208 bp
1,203 bp
958 bp
1,940,515 bp
l
25,960 bp
6,002 bp
24,842 bp
12,629 bp
5,122 bp
4,698 bp
1,809 bp
1,940,477 bp
SPAdes
m
n
o
p
q
r
Unicycler
s
10 bp
369 bp
460 bp
1,893,317 bp
t
6 bp
73 bp
874 bp
1,864,868
2,418 bp
u
v
1,894,156 bp
w
1,891,042 bp
2,418 bp
x
1,894,069 bp
