# Supplementary material for: Testing assembly strategies of Francisella tularensis genomes to infer an evolutionary conservation analysis of genomic structures
Source: BMC Genomics. 2021 Nov 14;22:822. doi: 10.1186/s12864-021-08115-x (PMC8590783; doi:10.1186/s12864-021-08115-x)
Supplement: Supplementary file 18 — Additional file 18: Supplementary Fig. 18. Affinity propagation clustering of all the 123 insertion sequences of FSC237 revealed 3 cluster. [file 12864_2021_8115_MOESM18_ESM.pdf]

Supplementary Figure 18: Affinity propagation clustering of all the 123 insertion sequences of FSC237 revealed 3 cluster (Number of samples= 123, Number of iterations = 169, Input preference = -2, Sum of similarities = -1.728201, Sum of preferences = -6, Net similarity = -7.728201, Number of clusters = 3

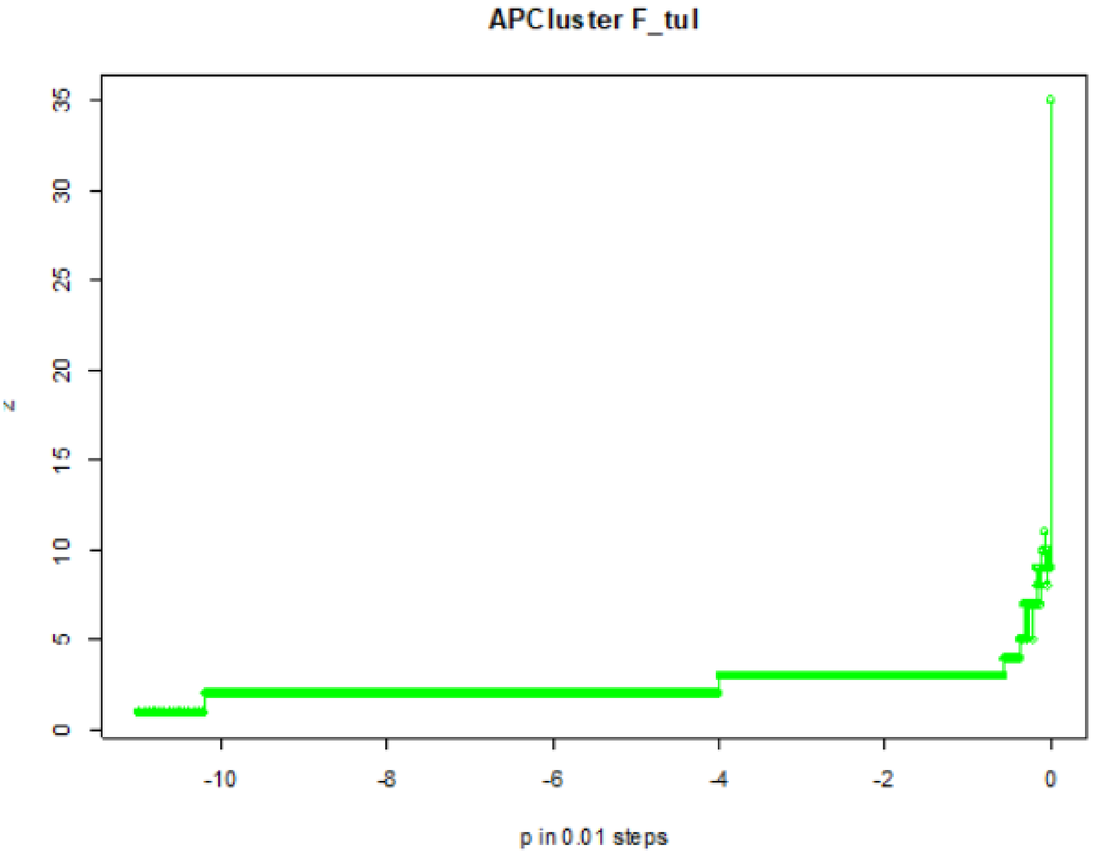

<u>Clusters:</u>

<u>Cluster 1, exemplar FSC237 - isftu1 14 CDS</u>:

FSC237 - IS1595 family transposase ISFtu3 CDS FSC237 - IS1595 family transposase ISFtu3 CDS 2, FSC237 - IS1595 family transposase ISFtu3 CDS 3 FSC237 - IS1595 family transposase ISFtu6 CDS, FSC237 - IS1595 family transposase ISFtu6 CDS 2 FSC237 - isftu1 2 CDS FSC237 - isftu1 3 CDS FSC237 - isftu1 5 CDS, FSC237 - isftu1 7 CDS FSC237 - isftu1 8 CDS FSC237 - isftu1 10 CDS FSC237 - isftu1 13 CDS FSC237 - isftu1 14 CDS, FSC237 - isftu1 17 CDS FSC237 - isftu1 18 CDS FSC237 - isftu1 20 CDS FSC237 - isftu1 23 CDS FSC237 - isftu1 25 CDS, FSC237 - isftu1 26 CDS FSC237 - isftu1 28 CDS FSC237 - isftu1 31 CDS FSC237 - isftu1 33 CDS FSC237 - isftu1 34 CDS, FSC237 - isftu1 37 CDS FSC237 - isftu1 39 CDS FSC237 - isftu1 40 CDS FSC237 - isftu1 42 CDS FSC237 - isftu1 44 CDS, FSC237 - isftu1 46 CDS FSC237 - isftu1 49 CDS FSC237 - isftu1 50 CDS FSC237 - isftu1 53 CDS FSC237 - isftu1 55 CDS, FSC237 - isftu1 56 CDS FSC237 - isftu1 58 CDS FSC237 - isftu1 61 CDS FSC237 - isftu1 62 CDS FSC237 - isftu1 64 CDS, FSC237 - isftu1 65 CDS FSC237 - isftu1 67 CDS FSC237 - isftu1 69 CDS FSC237 - isftu1 71 CDS FSC237 - isftu1 72 CDS, FSC237 - isftu1 75 CDS FSC237 - isftu1 77 CDS FSC237 - isftu1 78 CDS FSC237 - isftu1 80 CDS FSC237 - isftu1 82 CDS, FSC237 - isftu1 84 CDS FSC237 - isftu1 86 CDS FSC237 - isftu1 89 CDS FSC237 - isftu1 91 CDS FSC237 - isftu1 93 CDS, FSC237 - isftu1 95 CDS FSC237 - isftu1 96 CDS,

Cluster 2, exemplar FSC237 - isftu1 70 CDS:,

FSC237 - isftu1 1 CDS FSC237 - isftu1 4 CDS FSC237 - isftu1 6 CDS FSC237 - isftu1 9 CDS FSC237 - isftu1 11 CDS, FSC237 - isftu1 12 CDS FSC237 - isftu1 15 CDS FSC237 - isftu1 16 CDS FSC237 - isftu1 19 CDS FSC237 - isftu1 21 CDS, FSC237 - isftu1 22

CDS FSC237 - isftu1 24 CDS FSC237 - isftu1 27 CDS FSC237 - isftu1 29 CDS FSC237 - isftu1 30 CDS, FSC237 - isftu1 32 CDS FSC237 - isftu1 35 CDS FSC237 - isftu1 36 CDS FSC237 - isftu1 38 CDS FSC237 - isftu1 41 CDS, FSC237 - isftu1 43 CDS FSC237 - isftu1 45 CDS FSC237 - isftu1 47 CDS FSC237 - isftu1 48 CDS FSC237 - isftu1 51 CDS, FSC237 - isftu1 52 CDS FSC237 - isftu1 54 CDS FSC237 - isftu1 57 CDS FSC237 - isftu1 59 CDS FSC237 - isftu1 60 CDS, FSC237 - isftu1 63 CDS FSC237 - isftu1 66 CDS FSC237 - isftu1 68 CDS FSC237 - isftu1 70 CDS FSC237 - isftu1 73 CDS, FSC237 - isftu1 74 CDS FSC237 - isftu1 76 CDS FSC237 - isftu1 79 CDS FSC237 - isftu1 81 CDS FSC237 - isftu1 83 CDS, FSC237 - isftu1 85 CDS FSC237 - isftu1 87 CDS FSC237 - isftu1 88 CDS FSC237 - isftu1 90 CDS FSC237 - isftu1 92 CDS, FSC237 - isftu1 94 CDS FSC237 - isftu1 97 CDS FSC237 - IS4 family transposase ISFtu5 CDS

Cluster 3, exemplar FSC237 - isftu2 5 CDS:

FSC237 - IS982 family transposase ISFtu4 CDS FSC237 - IS982 family transposase ISFtu4 CDS 2, FSC237 - IS1595 family transposase ISFtu3 CDS 4 FSC237 - IS1595 family transposase ISFtu3 CDS 5 FSC237 - isftu2 1 CDS, FSC237 - isftu2 2 CDS FSC237 - isftu2 3 CDS FSC237 - isftu2 4 CDS FSC237 - isftu2 5 CDS FSC237 - isftu2 6 CDS, FSC237 - isftu2 7 CDS FSC237 - isftu2 8 CDS FSC237 - isftu2 9 CDS FSC237 - isftu2 10 CDS FSC237 - isftu2 11 CDS, FSC237 - isftu2 12 CDS FSC237 - isftu2 13 CDS FSC237 - isftu2 14 CDS FSC237 - isftu2 15 CDS FSC237 - isftu2 16 CDS, ,
